# *Klebsiella pneumoniae* from fuchka: insights into the genetic diversity, virulence, and antibiogram profile

**DOI:** 10.1128/aem.00996-26

**Published:** 2026-08-05

**Authors:** Bushra Benta Rahman Prapti, Md. Tanjir Ahmmed, Chinmoy Dev Nath, Aminur Rahman, Sakhyajit Saha Parijat, Partho Protim Acharjee, Jayedul Hassan, Md. Shafiqul Islam, Mahbubul Pratik Siddique

**Affiliations:** 1 Department of Microbiology and Hygiene, Faculty of Veterinary Science, Bangladesh Agricultural University 54492, Mymensingh, Bangladesh; Anses, Maisons-Alfort Laboratory for Food Safety, Maisons-Alfort, France

**Keywords:** street food, fuchka, *Klebsiella pneumoniae*, virulence, multi-drug resistance, biofilm

## Abstract

*Klebsiella pneumoniae* is an emerging pathogen of global concern due to its capacity to acquire antimicrobial resistance and virulence determinants, yet its presence in the food environment remains unexplored. This study investigated the prevalence, antimicrobial resistance, virulence genes, biofilm formation, and genomic characteristics of *K. pneumoniae* isolated from a widely consumed street food in Bangladesh. A total of 90 fuchka and related samples from different sources were collected and analyzed using culture-based methods, PCR confirmation, matrix-assisted laser desorption ionization time-of-flight assay, and 16S rRNA sequencing. Antibiotic sensitivity was determined using the disk diffusion method, and resistance- and virulence-associated genes were detected by PCR. Biofilm formation was quantified using a 96-well microtiter plate assay, and genetic diversity was assessed by enterobacterial repetitive intergenic consensus-PCR (ERIC-PCR) fingerprinting. Whole-genome sequencing (WGS) was performed on four representative isolates for in-depth genomic characterization. *K. pneumoniae* was recovered from 46.7% of samples. Among the isolates, 37.5% exhibited a multidrug-resistant (MDR) phenotype, with universal resistance to ampicillin. Virulence genes including *wab*G (90.5%) and *uge* (95.2%) were highly prevalent, whereas *rmp*A and *bfp* were absent. Capsular serotypes K3 and K5 were detected in 16.7% of isolates. The class 1 integron (*int*1) (83.3%) and *tet*A (56.7%) were the most frequently detected resistance determinants. Biofilm formation was observed in 31% of isolates and significantly associated with the MDR phenotype. WGS revealed clinically relevant sequence types (ST2262) phylogenomically related to human clinical strains. These findings indicate that fuchka may act as a reservoir for virulent and MDR *K. pneumoniae,* highlighting the urgent need for improved hygiene practices and integration of street food into the One Health antimicrobial surveillance framework.

**IMPORTANCE:**

The presence of multidrug-resistant (MDR) *Klebsiella pneumoniae* in ready-to-eat street foods poses a significant public health concern, particularly in low- and middle-income countries where informal food vending is widespread. Fuchka is consumed daily by diverse population groups in Bangladesh, including children and young adults, making it a potential route for silent transmission of clinically important pathogens. The detection of MDR strains harboring virulence factors, integrons, and biofilm-forming ability suggests an elevated risk of persistence, spread, and limited treatment options in case of infection. This study highlights street food as an overlooked reservoir in the One Health framework, bridging community environments and clinical settings. Improved surveillance, vendor hygiene training, and enforcement of food safety regulations are crucial to reduce the risk of dissemination of these high-risk strains.

## INTRODUCTION

*Klebsiella pneumoniae*, a member of the Enterobacteriaceae family, has transitioned from a relatively harmless commensal to one of the most resilient multidrug-resistant (MDR) pathogens of the 21st century (1). It is mostly associated with community-acquired and healthcare-associated infections, including pneumonia, urinary tract infections, septicemia, and liver abscesses (2). The World Health Organization (WHO) has classified it as a “critical priority” pathogen, underscoring the urgent need for research and the development of new antibiotics (3). The worldwide spread of *K. pneumoniae* infections, especially those caused by MDR strains, has raised significant concerns in the medical field (4). Such strains have been reported in various regions, including India, Pakistan, the United Kingdom, the United States, Canada, and Japan (5), and are associated with increased morbidity and mortality, particularly in vulnerable groups such as hospitalized patients (6).

A major driver of this clinical threat is the organism’s remarkable capacity to readily acquire antimicrobial resistance (AMR) determinants, particularly extended-spectrum β-lactamases (ESBLs) and carbapenemases, through a vast arsenal of mobile genetic elements (7). Consequently, *Klebsiella* has already developed resistance to many advanced and critically important antibiotics like cephalosporins and carbapenems, which are often used as last-resort treatments for severe infections (8). Beyond resistance, the pathogenicity of *K. pneumoniae* has been enhanced by a variety of virulence factors, including adhesins, capsule-associated determinants, siderophore production, and biofilm-forming capacity (2). Hypervirulent strains, characterized by increased capsular production and hyper-mucoviscosity, can cause infection even in otherwise healthy individuals (9, 10). Capsular serotypes such as K1, K2, K54, K57, K20, and K5 are strongly linked to hypervirulence and are frequently implicated in serious clinical conditions, including liver abscesses and septicemia (11, 12). Biofilm formation further contributes to environmental persistence and resistance to antimicrobial agents (13). Key biofilm-associated virulence genes include capsular polysaccharide (CPS) cluster genes (*cps*D, *tre*C, *wab*G, *wca*G, *wzc*, k2A, and *wzy*K2), type III fimbriae genes (*mrk*A and *mrk*D), and quorum-sensing gene (*lux*S), *rmp*A (regulating the mucoid phenotype), allS (involved in allantoin metabolism), endotoxin-related genes (*wab*G, *uge*, *wca*G), iron acquisition genes (*iuc*B, *iro*NB, *ybt*A, *kfu*BC), and *fim*H (type I fimbrial adhesin) (12, 14).

Foodborne exposure represents an increasingly recognized but unexplored pathway for the transmission of *K. pneumoniae* (15). Ready-to-eat foods are of particular concern, as they are frequently prepared under suboptimal hygienic conditions and consumed without further heating (16). In densely populated urban settings, street food acts as a bridge connecting environmental contamination and food handling practices (17). Fuchka is one of the most popular street foods in Bangladesh and is consumed daily by people of all age groups (18). Its preparation involves extensive manual handling, use of raw ingredients, and untreated water, creating multiple opportunities for microbial contamination (19). Although resistant Enterobacteriaceae have occasionally been reported in street foods (20, 21), detailed investigations focusing specifically on *K. pneumoniae* in traditional foods such as fuchka remain scarce (22). Studies from Europe, Asia, and Africa have detected *K. pneumoniae* in various RTE foods, including salads, cheese, salami, and panipuri (23–26).

In this study, the first detailed molecular and phenotypic characterization of *K. pneumoniae* strains recovered from fuchka sold in Mymensingh, Bangladesh, has been provided. Moreover, the co-occurrence of virulence genes and drug resistance traits of *K. pneumoniae* has been explored, along with their possible impact on public health. By combining microbiological, genetic, and public health approaches, this research highlights the need to include informal food sectors in national plans to combat antimicrobial resistance. It further emphasizes that foodborne *K. pneumoniae* should not be considered a rare contaminant but rather an important indicator of public health risk in the age of antibiotic resistance.

## RESULTS

### Prevalence of *Klebsiella pneumoniae* and serotype detection

**Fig 1:**
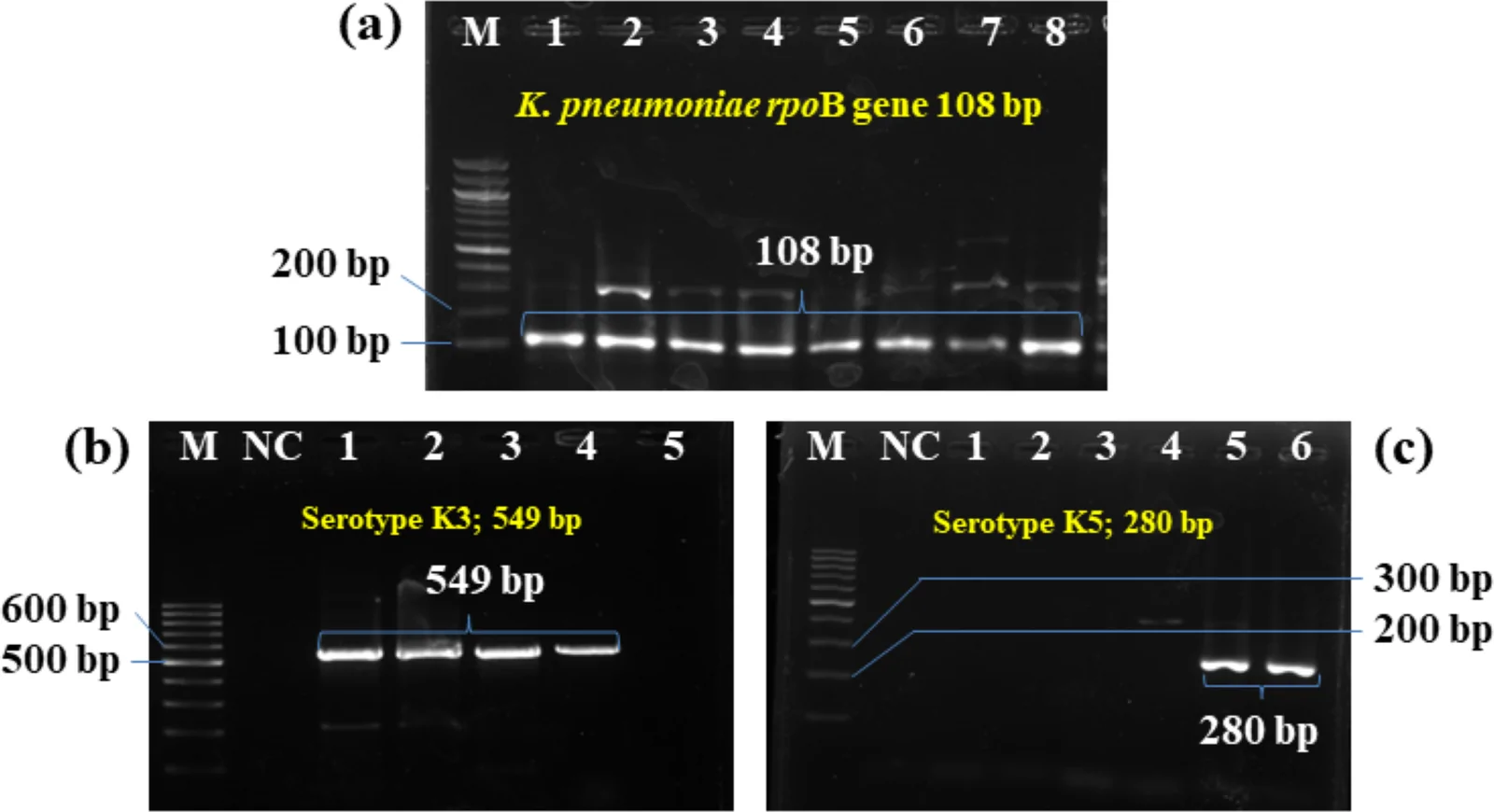
PCR confirmation of *K. pneumoniae* and serotype determination. (**a**) *rpo*B gene-based PCR detection of *K. pneumoniae* with consistent amplicon size of 108 bp; (**b**) PCR detection of serotype K3 from fuchka samples with amplicon size of 549 bp; (**c**) serotype K5 detection with PCR band at 280 bp. M, 100 bp molecular marker; bp, base pair; NC, negative control. PCR bands for positive isolates are indicated in the figures.

Out of 90 samples analyzed, *Klebsiella pneumoniae* was detected in 42 samples, yielding an overall prevalence of 46.7% (Fig. 1a). The distribution of the isolates varied by source, with the highest number recovered from street vendors (*n* = 18), followed by fuchka-selling shops (*n* = 12), restaurants (*n* = 6), and fuchka delivery services (*n* = 6). Serotype identification detected four isolates as K3 and three isolates as K5 (Fig. 1b and c). None of the isolates belonged to serotype K1, K2, or K20.

### Distribution of virulence genes

**Fig 2:**
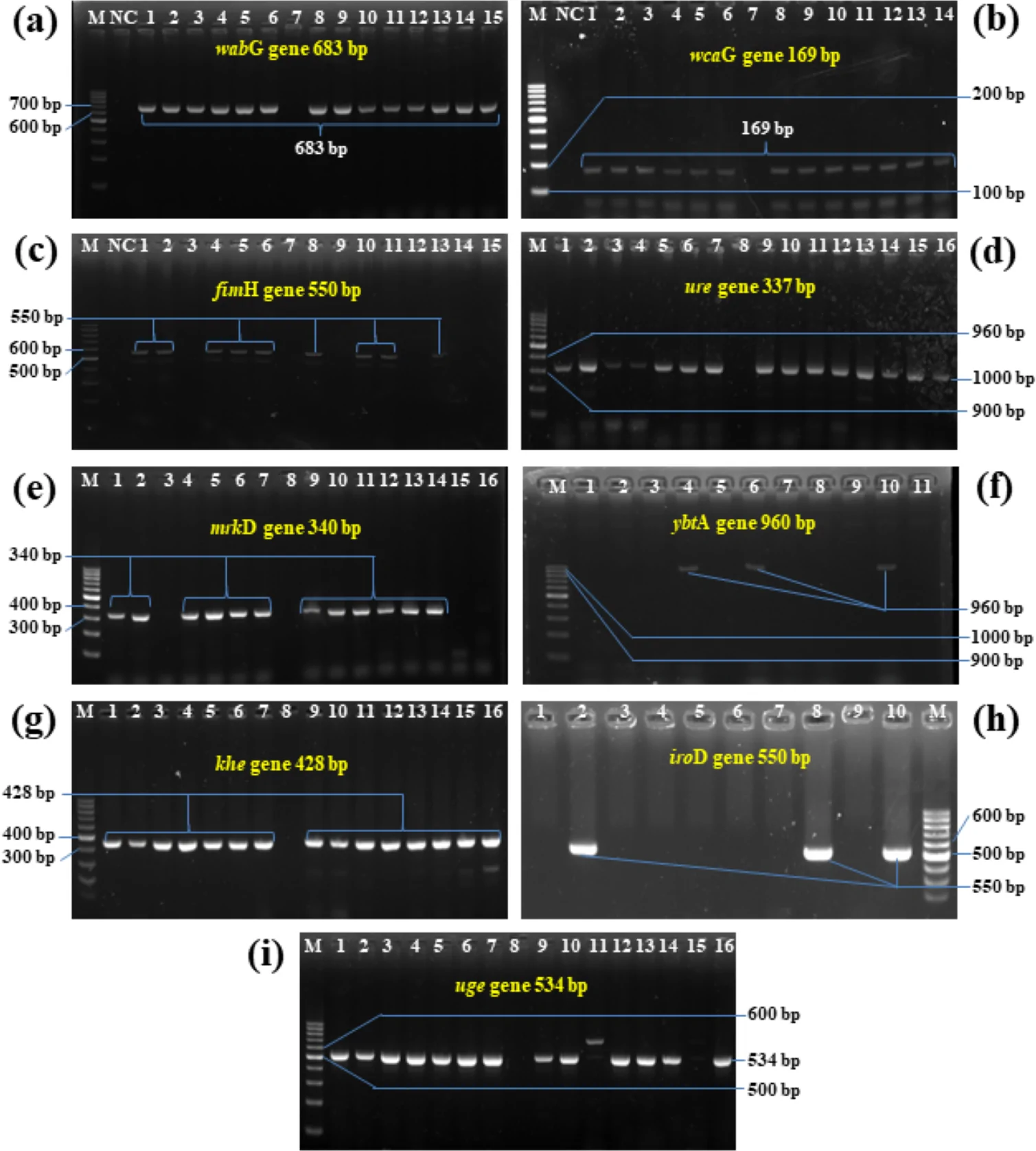
PCR detection of virulence genes from fuchka isolates of *K. pneumoniae*. Specific virulence-associated genes and consistent and specific band sizes are mentioned in the respective figures. M, 100 bp molecular marker; bp, base pair; NC, negative control.

**Fig 3:**
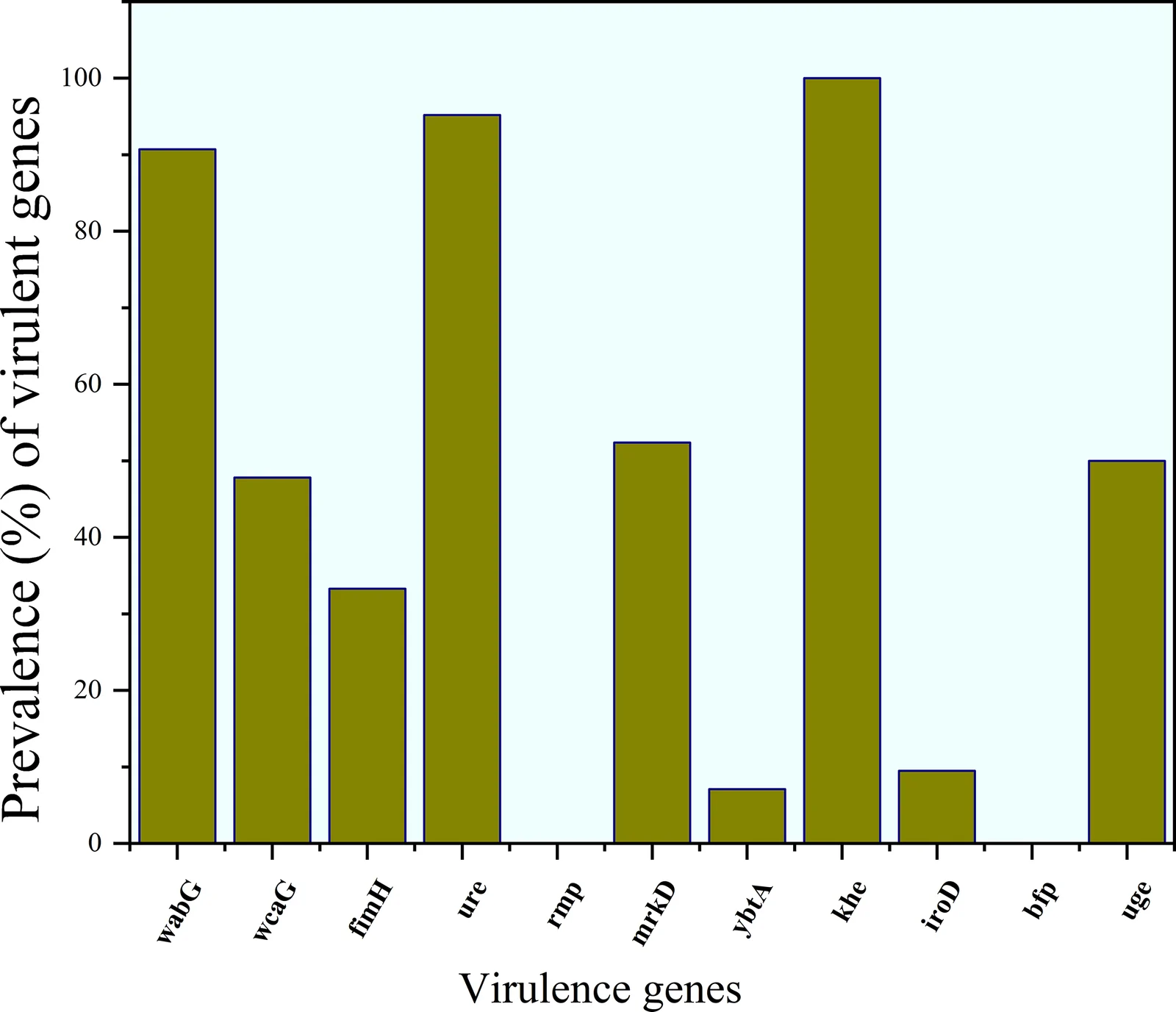
Overall prevalence (percentage) of virulence genes in *K. pneumoniae* isolated from fuchka samples.

The presence of virulence-associated factors was confirmed by PCR and illustrated in Fig. 2. A higher prevalence was observed for endotoxin-related genes (*wab*G, *uge*, and *wca*G), with *wab*G found in 90.5% of isolates, *wca*G in 47.6%, and *uge* in 95.2%. In contrast, the prevalence of siderophore-related genes was markedly lower, with *ybt*A and *aerobactin* detected in 7.1% and 9.5% of isolates, respectively. None of the isolates carried the (regulator of mucoid phenotype) *rmp*A or *bfp* genes. Detailed frequencies of all virulence genes are presented in Fig. 3.

**Fig 4:**
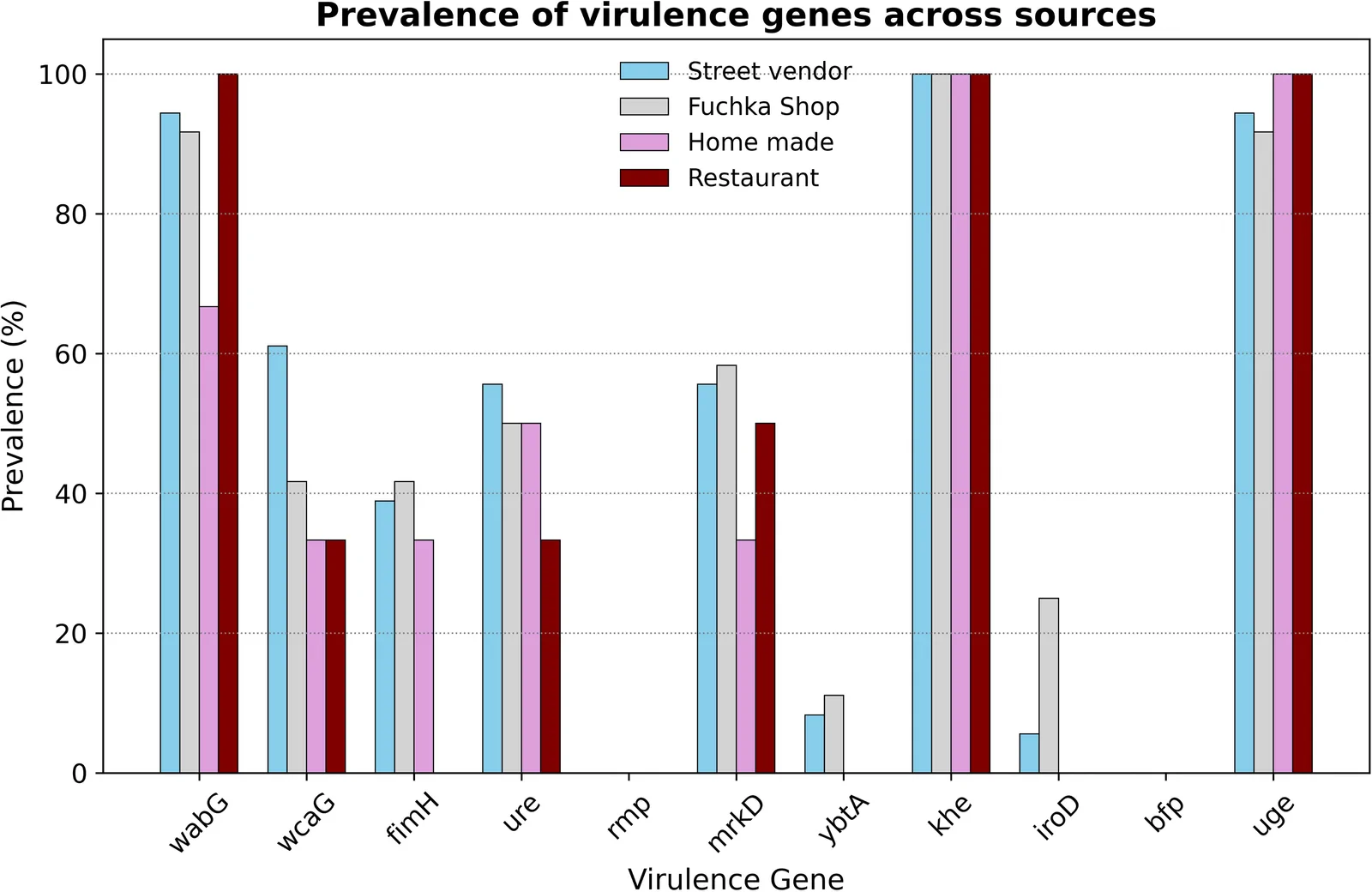
Sample-wise prevalence (percentage) of virulence genes in fuchka isolates of *K. pneumoniae*.

Moreover, the distribution of virulence genes varied across the samples obtained from different sources (Fig. 4). *wab*G and *uge* were highly prevalent, particularly among samples from restaurants (100%) and street vendors (94.4%). In contrast, *wca*G exhibited moderate distribution, ranging from 61.1% in street vendor samples to 33.3% in restaurant and homemade samples. *ure* and *mrk*D were detected in approximately half of the isolates from all sources. *ybt*A and *aerobactin* were less frequently detected, only in a few isolates from street vendors (8.3% and 5.6%, respectively) and fuchka shops (11.1% and 25%, respectively), and were completely absent in restaurant and homemade fuchka samples.

### Distribution of virulence genes among different *K. pneumoniae* serotypes

Among the *K. pneumoniae* isolates, most were non-typeable (83.3%), with only a small portion of isolates identified as K3 (9.5%) and K5 (7.1%). All K3 and K5 isolates consistently carried *wab*G and *khe* genes (100%). In K3 isolates, *fimH*, *ure*, *mrkD*, and *wcaG* were detected in 75% of isolates, while *aerobactin* occurred less frequently. K5 isolates showed lower variability, with *mrk*D being the most frequently observed gene (66.7%). *khe* was universally present (100%) among the non-typeable isolates, followed by *wab*G and *uge* (97.1% each) (Table 1).

**TABLE 1:** Distribution of virulence genes among *Klebsiella pneumoniae* serotypes^*a*^

Serotype	Number of isolates (percentage)	Virulence genes (frequency and percentage within serotype)
K3	4 (9.5%)	*wab*G, *khe*, *uge*: 4/4 (100%)
		*fim*H, *ure*, *mrk*D, *wca*G: 3/4 (75%)
		*aerobactin*: 1/4 (25%)
K5	3 (7.1%)	*wab*G, *khe*, *uge*: 3/3 (100%)
		*mrk*D: 2/3 (66.7%)
		*fim*H, *aerobactin*: 1/3 (33.3%) each
Non-typeable	35 (83.3%)	*khe*: 34/35 (97.1%) each
		*wab*G, *uge*: 24/35 (68.6%) each
		*wca*G: 17/35 (48.6%) each
		*fim*H: 13/35 (37.1%) each
		*aerobactin*: 3/35 (8.6%)
		*ybt*A: 2/35 (5.7%)

^*a*^ Percentages based on small sample sizes should be interpreted with caution.

### Antimicrobial susceptibility profile, MAR index, and prevalence of MDR isolates

**Fig 5:**
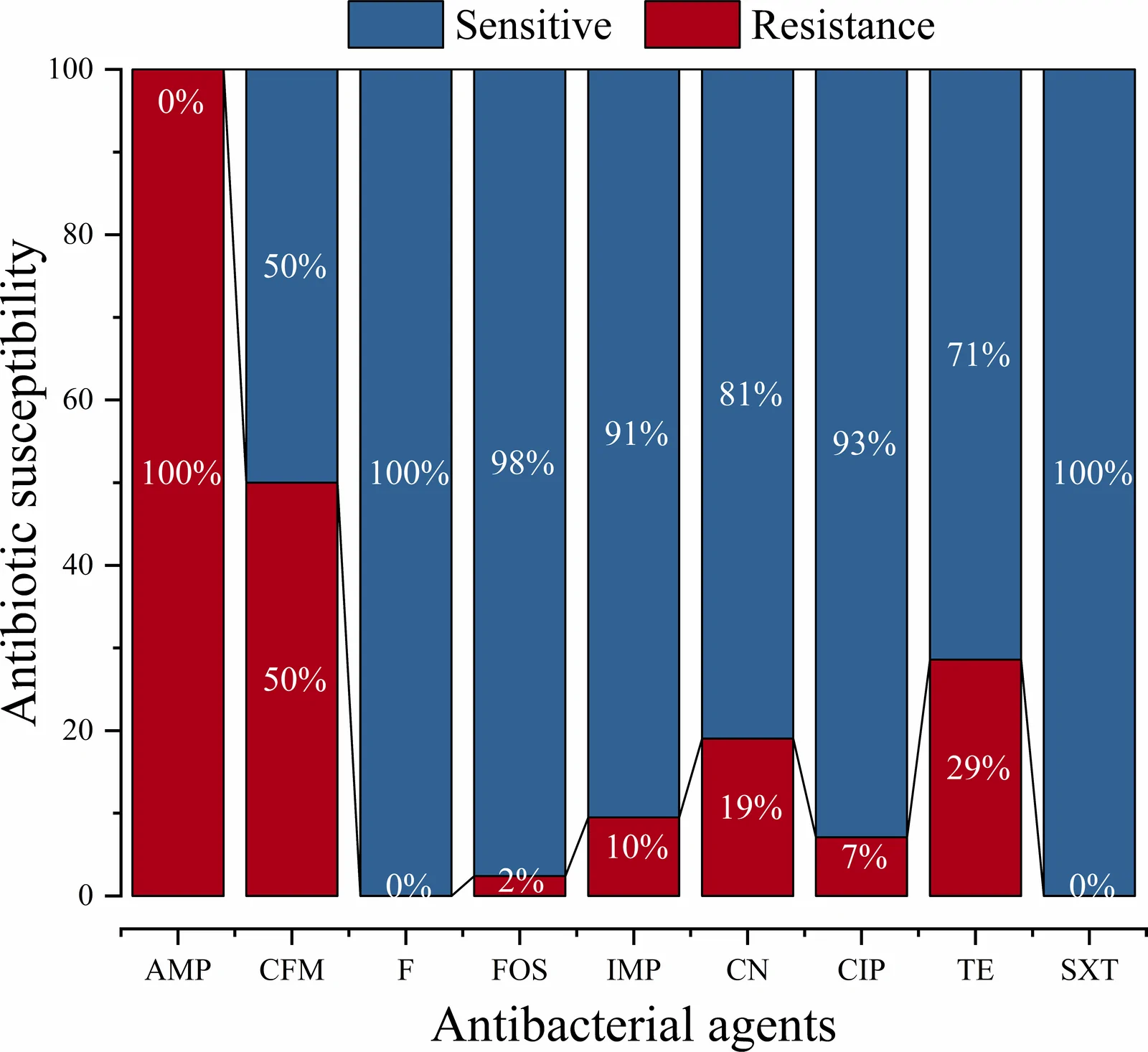
Antibiotic susceptibility pattern of *K. pneumoniae* isolates.

**Fig 6:**
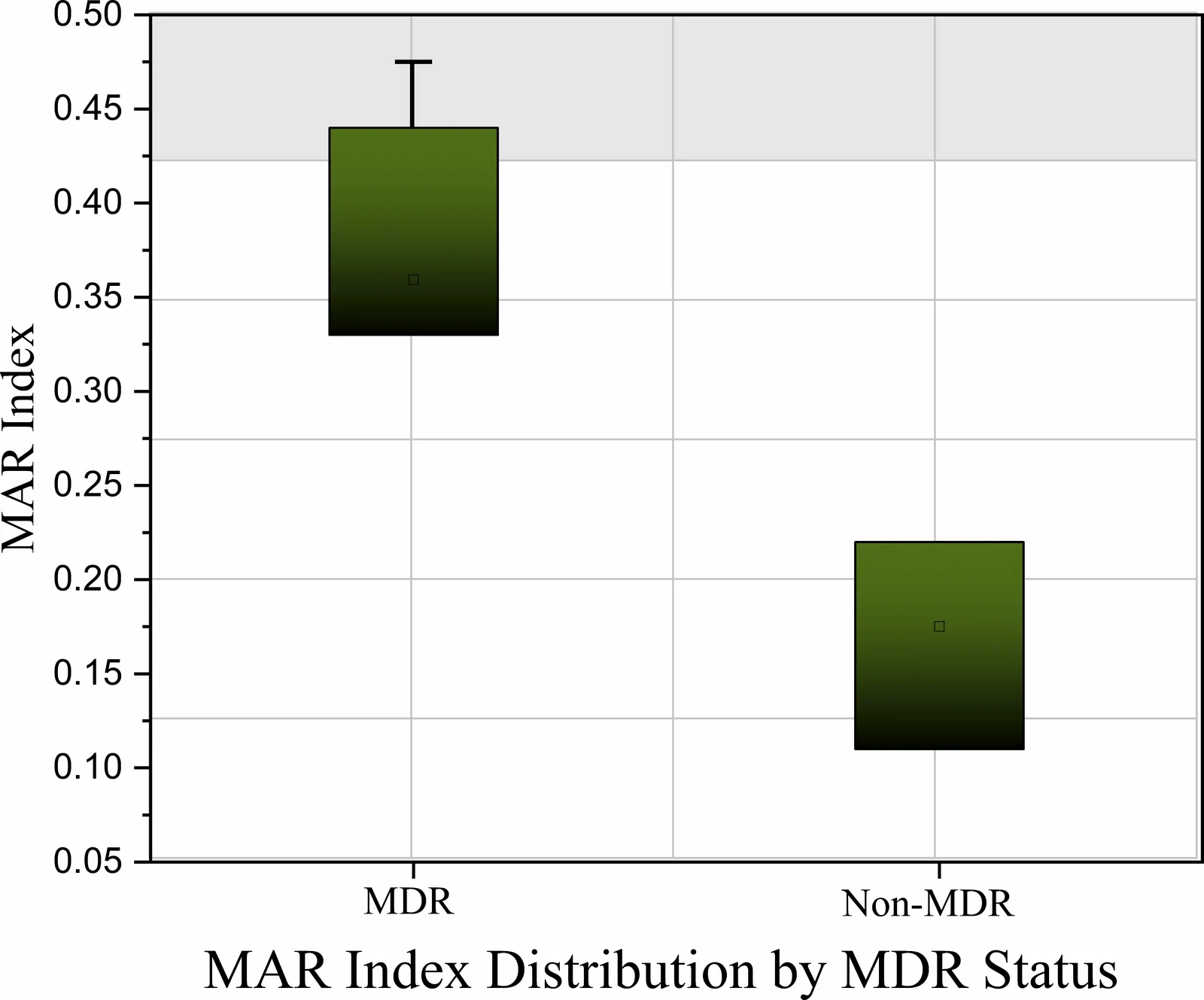
Distribution of the MAR index among the MDR and drug-resistant isolates.

All isolates were resistant to ampicillin. High resistance was observed for cefixime, with 52.4% (*n* = 22/42). In contrast, complete susceptibility (100%) was noted for nitrofurantoin and trimethoprim-sulfamethoxazole, while fosfomycin showed a susceptibility rate of 97.6%. Gentamicin demonstrated substantial activity (80.9% sensitive), and ciprofloxacin and imipenem exhibited high efficacy. Tetracycline resistance was observed in 28.6% of isolates. The multiple antibiotic resistance (MAR) index ranged from 0.11 to 0.44. MDR (resistance to ≥3 antibiotic classes) was detected in 35.7% of isolates, with a higher MAR index of 0.33–0.44 compared to non-MDR isolates (0.11 and 0.22) (Fig. 5 and 6).

### Prevalence of biofilm-producing *K. pneumoniae* and association of biofilm formation with MAR index and MDR phenotype

**Fig 7:**
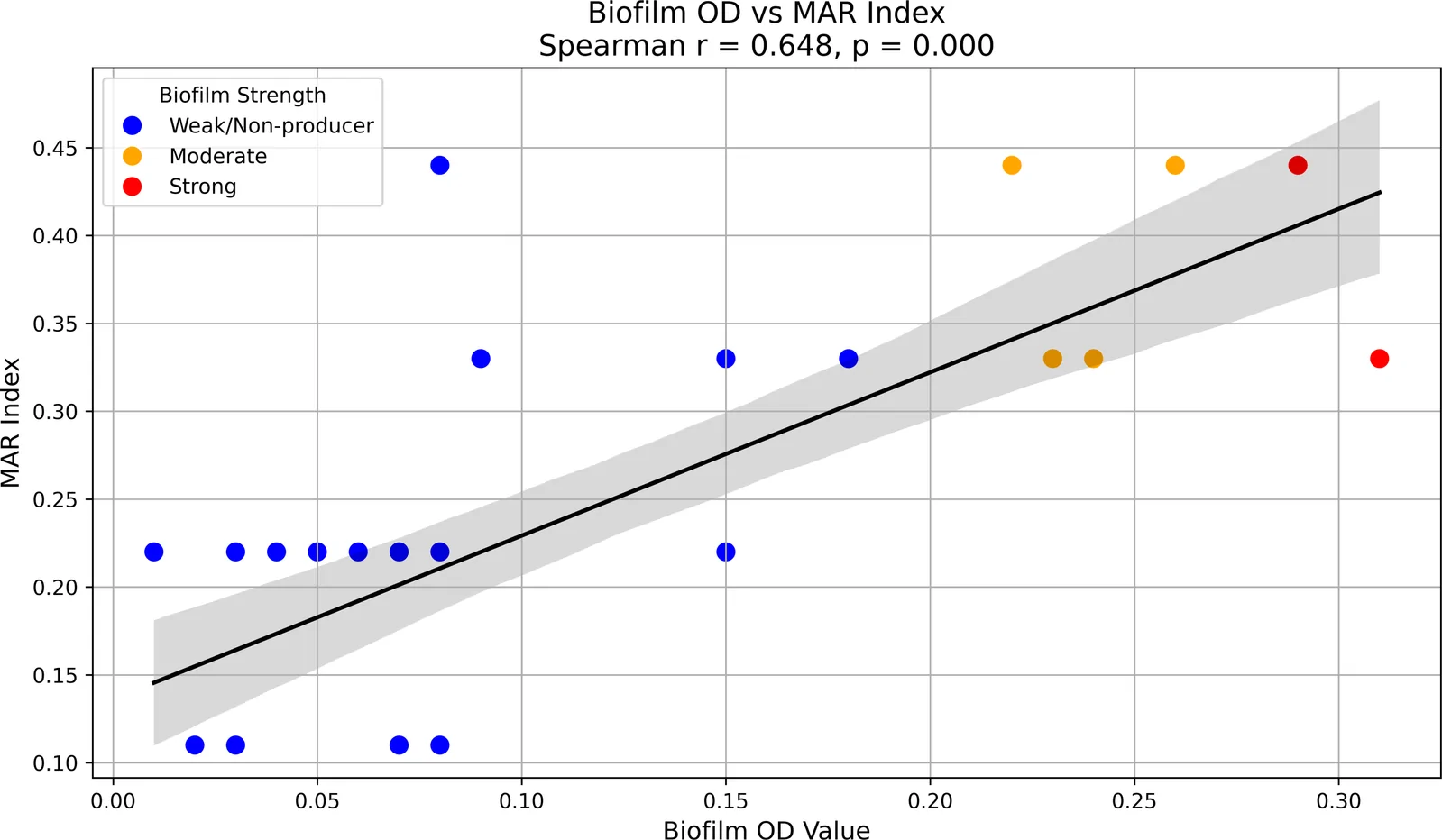
Correlation between the MAR index and biofilm OD value. Correlation analysis (by Spearman’s rank) revealed a strong association between biofilm OD value and MAR index (ρ = 0.648, *P* < 0.01).

Of 42 isolates, 13 (31%) were biofilm producers: 2 (15.4%) strong, 6 (46.2%) moderate, and 5 (38.5%) weak. Spearman’s rank correlation revealed a significant strong association between biofilm optical density (OD) value and MAR index (ρ = 0.648, *P* < 0.01), indicating that isolates with higher biofilm-forming ability tended to exhibit greater MAR indices (Fig. 7). Chi-square analysis confirmed a statistically significant association between biofilm-forming capacity and MDR phenotype (χ² = 27.834, *df* = 6, *P* < 0.001). MDR isolates were more frequently observed among strong and moderate biofilm producers compared with weak and non-biofilm producers (Table 2).

**TABLE 2:** Association between biofilm formation and multidrug resistance (MDR) phenotype in *K. pneumoniae*

Biofilm category	Non-MDR (*n*)	MDR (*n*)	Total (*n*)	χ^2^ value	*P*-value
Non	26	3	29	27.83	<0.001
Weak	1	4	5		
Moderate	0	6	6		
Strong	0	2	2		
Total	27	15	42		

### Prevalence of antibiotic resistance genes

**Fig 8:**
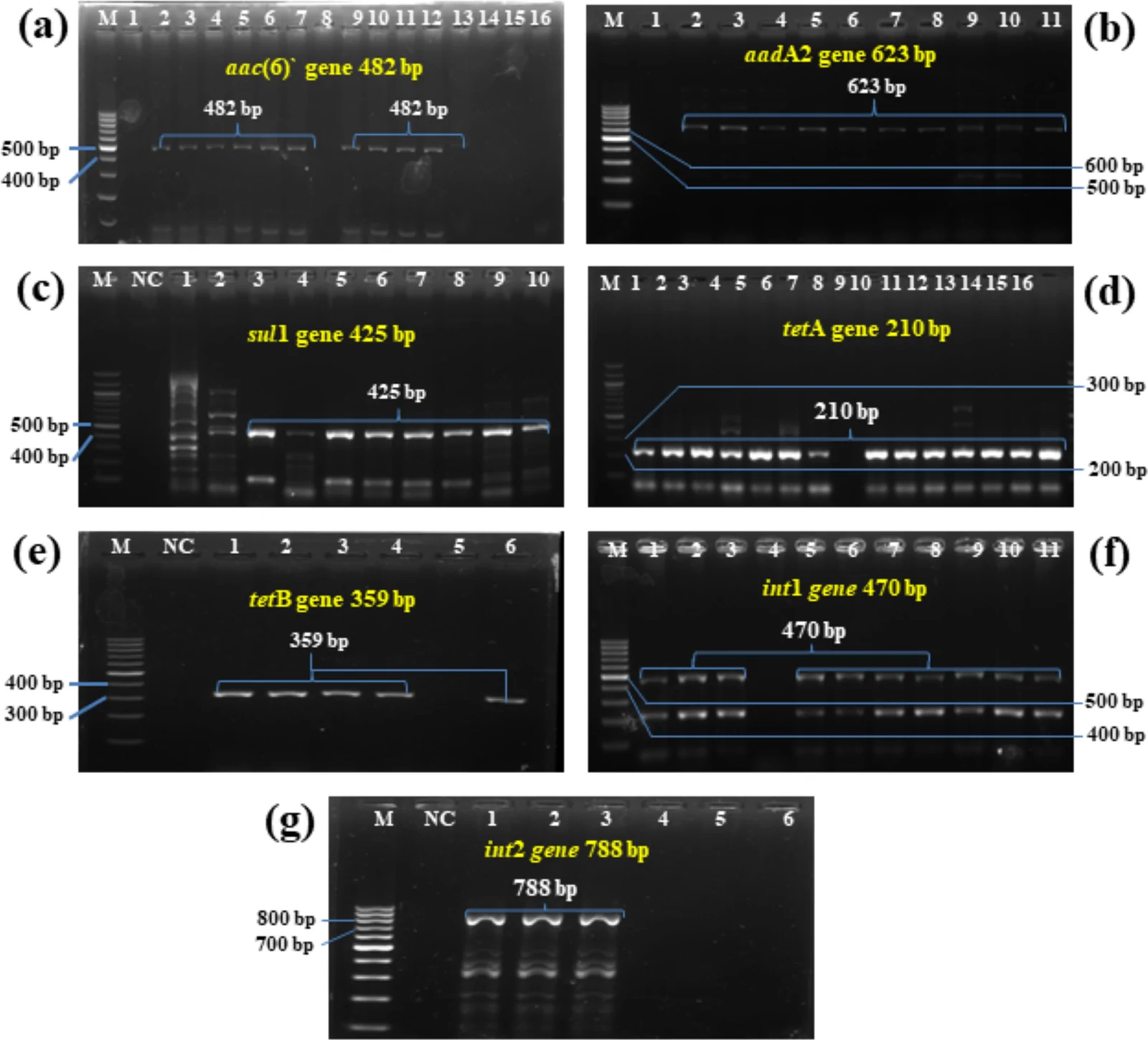
Detection of antibiotic resistance genes of isolated *K. pneumoniae* by PCR. Specific resistance genes and band sizes are mentioned on the respective figures. M, 100 bp molecular marker; bp, base pair; NC, negative control.

**Fig 9:**
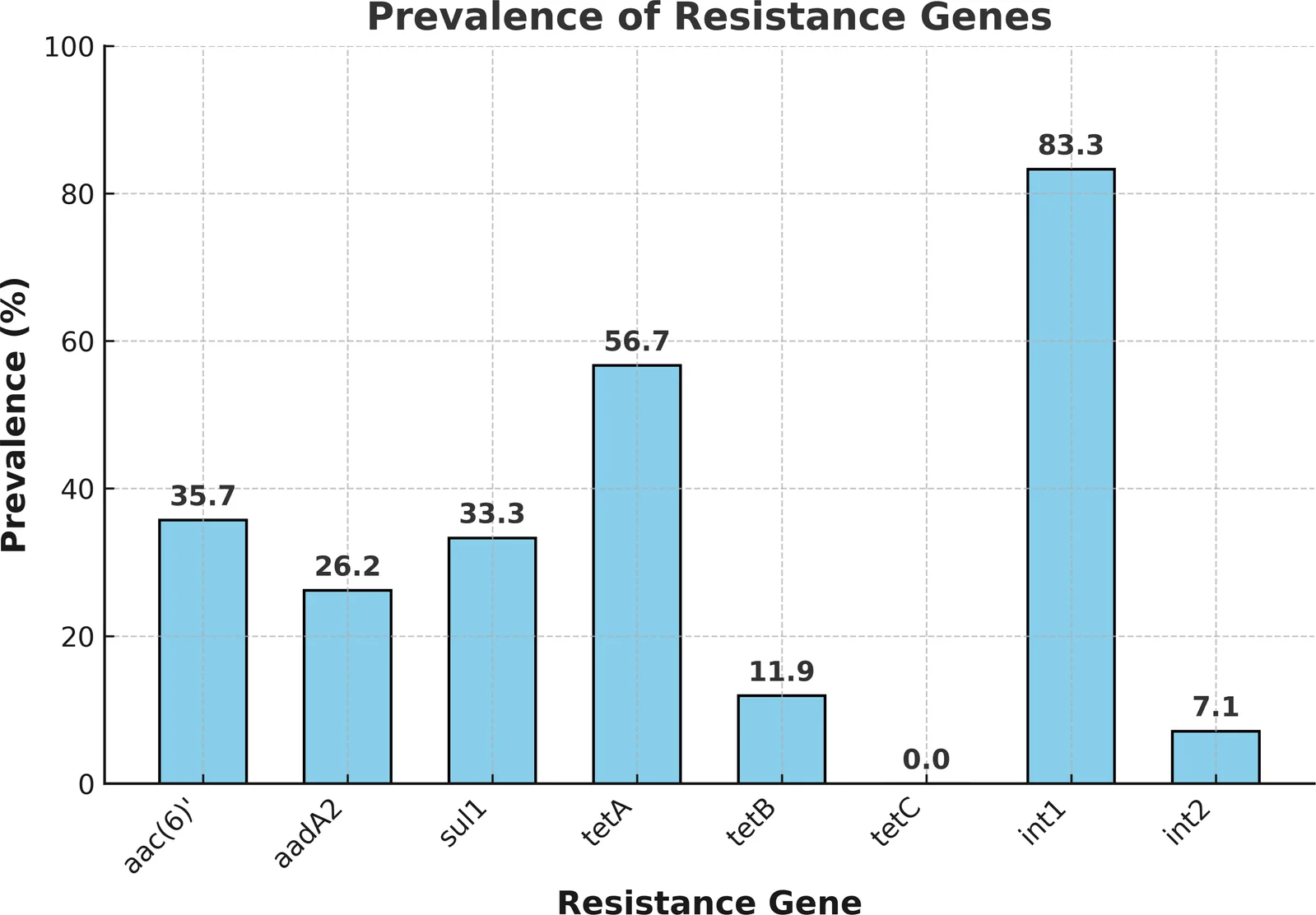
Overall prevalence (percentage) of resistance genes of the isolated *K. pneumoniae*.

**Fig 10:**
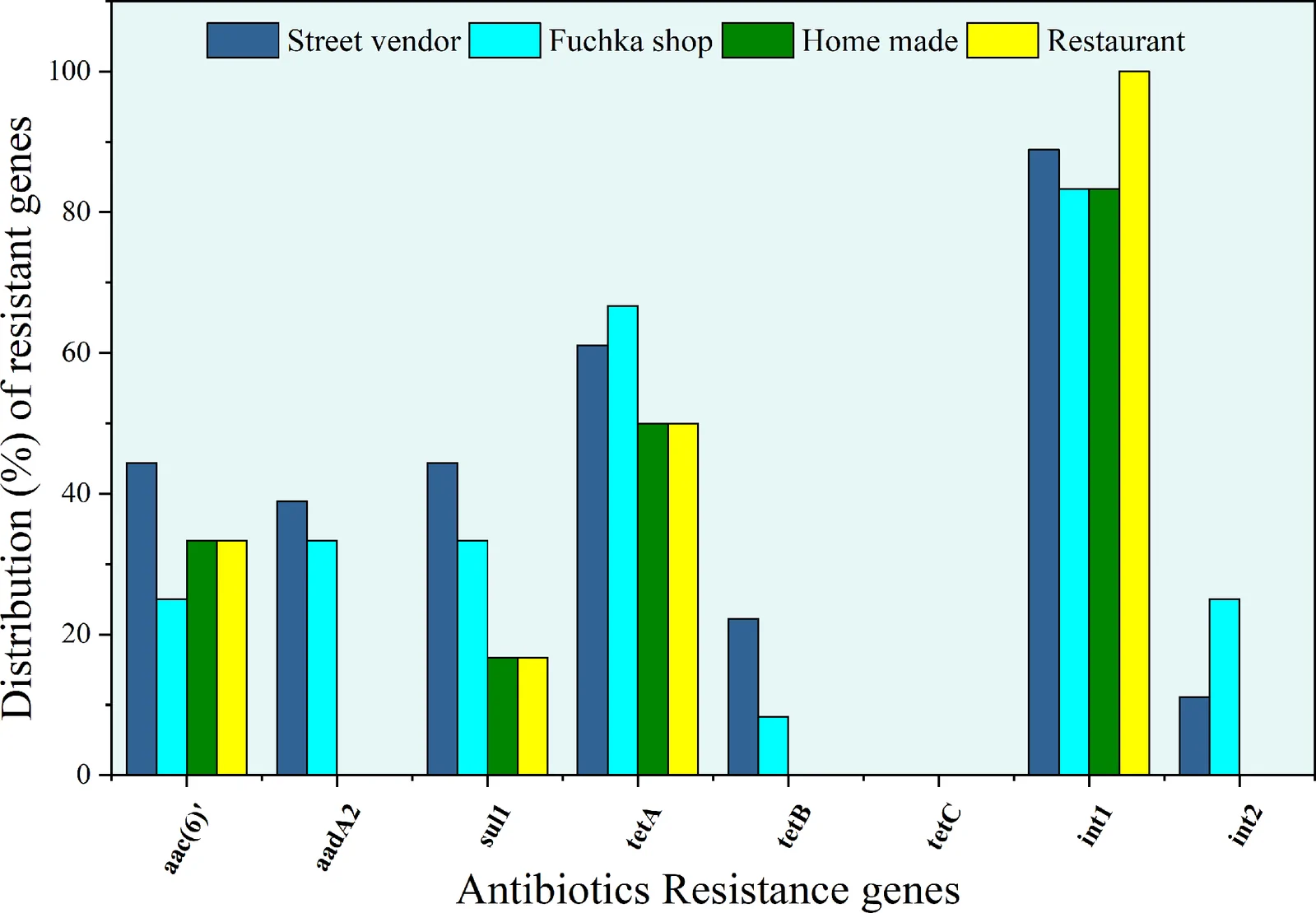
Sample-wise prevalence of resistance genes of fuchka isolates of *K. pneumoniae*.

All isolates harbored at least one of the tested resistance genes, and the presence of resistance genes was determined by PCR (Fig. 8). The class 1 integron (*int*1) and (*tet*A) were the most frequently detected determinants (83.3% and 56.7%, respectively), followed by *aac*6′ (35.7%), *sul*1 (33.3%), and *tet*B (11.9%), while the *tet*C gene was absent. *Int*1 prevalence reached 100% in restaurant-derived isolates. *aac*6′, *aad*A2, and *sul*1 were most common among street vendor isolates (Fig. 9 and 10).

### Association and co-occurrence of virulence and resistance genes

Analysis of co-occurrence patterns revealed that most virulence resistance gene associations were statistically nonsignificant (Table 3). However, significant associations included *wca*G-*sul*1 (50% co-occurrence, *P* = 0.02), *fim*H-*aac*6′ (33.3% co-occurrence, *P* = 0.04), and *ybt*A-*sul*1 (100% co-occurrence, *P* = 0.01). The integrase gene (*int*1) frequently co-occurred with most of the virulence genes, with a prevalence rate exceeding 90%, and *tet*A frequently co-occurred with *wca*G (75.0%) and *ybt*A (100%), though most were non-significant.

**TABLE 3:** Co-occurrence analysis of virulence and resistance genes among *Klebsiella pneumoniae* isolates^*a*^

Virulence gene	Resistance gene	Co-occurrence (%)	*P*-value	Virulence gene	Resistance gene	Co-occurrence (%)	*P*-value
*wab*G	*aac*6*′*	36.8%	0.64	*wca*G	*aac*6*′*	30.0%	0.46
	*aad*A2	26.3%	0.96		*aad*A2	25.0%	0.87
	*sul*1	34.2%	0.71		*sul*1	**50.0**%	**0.02**
	*tet*A	60.5%	0.68		*tet*A	**75.0%**	**0.05**
	*tet*B	13.2%	0.44		*tet*B	15.0%	0.55
	*tet*C	0%	–		*tet*C	0%	–
	*int*1	94.7%	0.15		*int*1	95.0%	0.60
	*int*2	5.4%	0.26		*int*2	10.0%	0.49
*fim*H	*aac*6*′*	33.3%	**0.04**	*ure*	*aac*6*′*	35.7%	0.33
	*aad*A2	26.2%	0.80		*aad*A2	26.2%	0.29
	*sul*1	33.3%	0.64		*sul*1	33.3%	0.19
	*tet*A	59.5%	0.82		*tet*A	59.5%	0.11
	*tet*B	11.9%	0.50		*tet*B	11.9%	0.63
	*tet*C	0%	–		*tet*C	0%	–
	*int*1	92.9%	0.20		*int*1	92.9%	0.55
	*int*2	7.1%	1.00		*int*2	7.1% (3/42)	0.55
*mrk*D	*aac*6*′*	35.7%	0.93	*ybt*A	*aac*6*′*	0%	0.18
	*aad*A2	26.2%	0.87		*aad*A2	0%	0.28
	*sul*1	33.3%	0.13		*sul*1	100%^*b*^	**0.01**
	*tet*A	59.5%	0.23		*tet*A	100%	0.14
	*tet*B	11.9%	0.02		*tet*B	33.3%	0.23
	*tet*C	0%	–		*tet*C	0%	–
	*int*1	92.9%	0.05		*int*1	100%	0.62
	*int*2	7.1%	0.60		*int*2	33.3%	0.06
*khe*	*aac*6*′*	37.5%	–	*aerobactin*	*aac*6*′*	35.7%	0.86
	*aad*A2	26.2%	–		*aad*A2	26.2%	0.67
	*sul*1	33.3%	–		*sul*1	33.3%	0.93
	*tet*A	59.5%	–		*tet*A	59.5%	0.09
	*tet*B	11.9%	–		*tet*B	11.9%	0.38
	*tet*C	0%	–		*tet*C	0%	–
	*int*1	92.9%	–		*int*1	92.9%	0.61
	*int*2	7.1%	–		*int*2	0%	0.12
*uge*	*aac*6*′*	37.5%	0.28				
	*aad*A2	27.5%	0.38				
	*sul*1	32.5%	0.60				
	*tet*A	60.0%	0.77				
	*tet*B	12.5%	0.59				
	*tet*C	0%	–				
	*int*1	92.5%	0.68				
	*int*2	7.5%	0.68				

^*a*^ –, not applicable; no statistical analysis was performed because the *tet*C gene was not detected in any isolate. Boldface indicates statistically significant associations (*P* ≤ 0.05).

^*b*^ Percentages based on a small number of isolates should be interpreted with caution.

### ERIC-PCR and molecular typing

**Fig 11:**
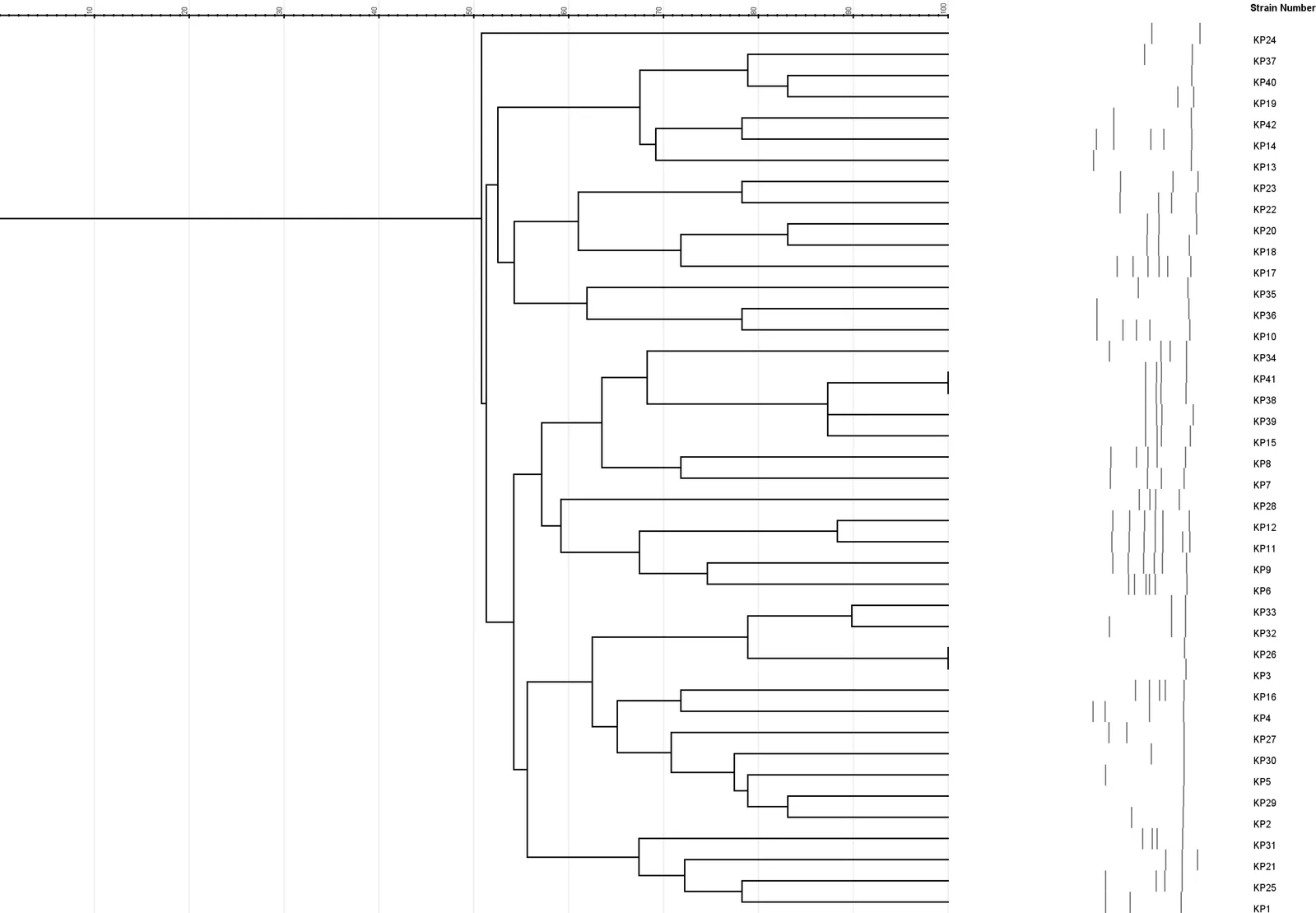
ERIC-PCR dendrogram showing the genetic relatedness among 42 isolates based on UPGMA clustering.

At the 80% similarity, the enterobacterial repetitive intergenic consensus (ERIC)-PCR dendrogram of 42 isolates was divided into seven distinct clusters along with a few singletons (Cluster I, 11 isolates; Cluster II, 7 isolates; Cluster III, 6 isolates; Cluster IV, 5 isolates; Cluster V, 4 isolates; Cluster VI, 3 isolates; Cluster VII, 2 isolates; four singletons) (Fig. 11), highlighting considerable genotypic heterogeneity within the collection.

### 16S rRNA sequence analysis

**Fig 12:**
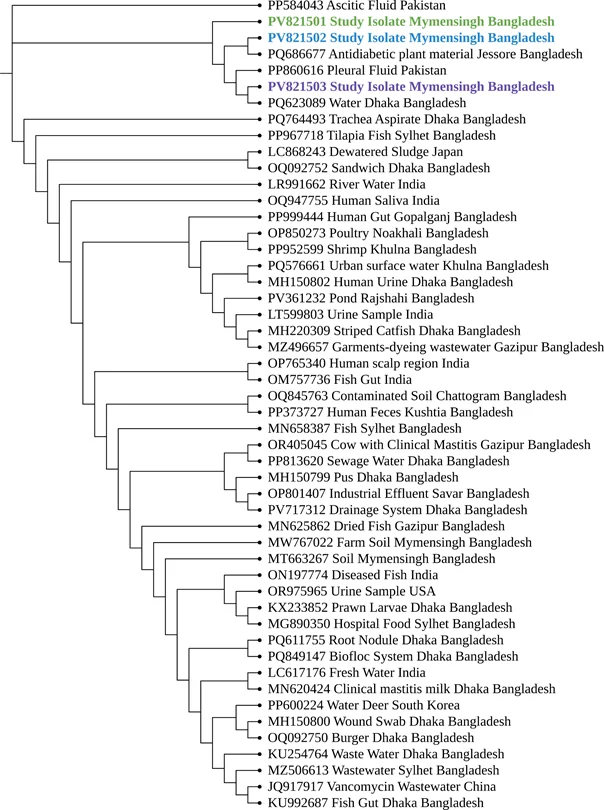
Phylogenetic tree showing the evolutionary relationship among the study isolates and the reference isolates found in NCBI GenBank. Study isolates are indicated in colored bold text.

Phylogenetic analysis of 16S rRNA gene sequences from three isolates placed the study strains in a distinct clade with reference strains PQ686677, PP860616, and PQ623089, indicating close genetic relatedness to Pakistani and Bangladeshi isolates currently circulating in the region (Fig. 12).

### Whole-genome sequencing (WGS): genomic characterization and comparative analysis

**Fig 13:**
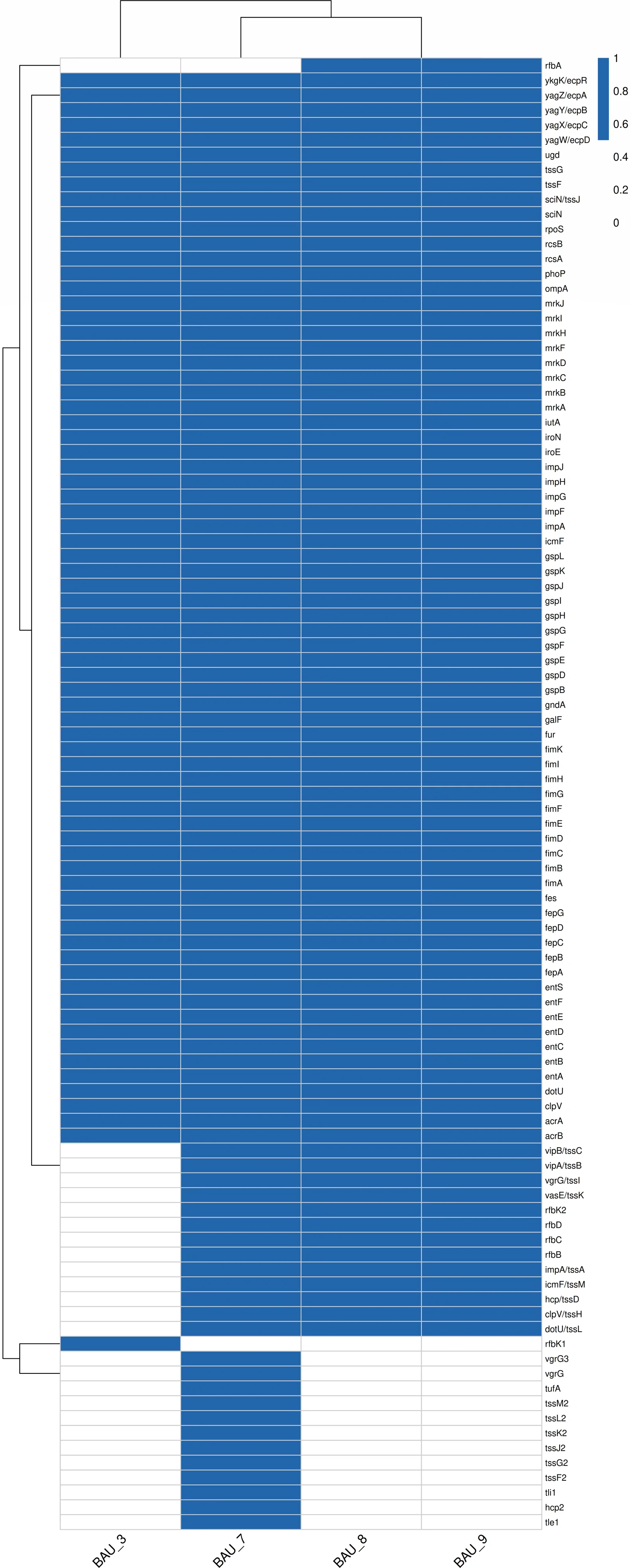
Representation of the virulence gene repertoire of the *K. pneumoniae* genome sequences described in this study. Heatmap showing the distribution of virulence-associated genes identified in isolates BAU_3 (MPS_KP(F)_3), BAU_7 (MPS_KP(DW)_7), BAU_8 (MPS_KP(S)_8), and BAU_9 (MPS_KP(HW)_9) using the Virulence Factor Database (VFDB). Virulence genes were detected using DIAMOND BLASTP with a stringent threshold of ≥90% query and subject coverage. Blue indicates presence, while white indicates absence of the corresponding virulence gene.

**Fig 14:**
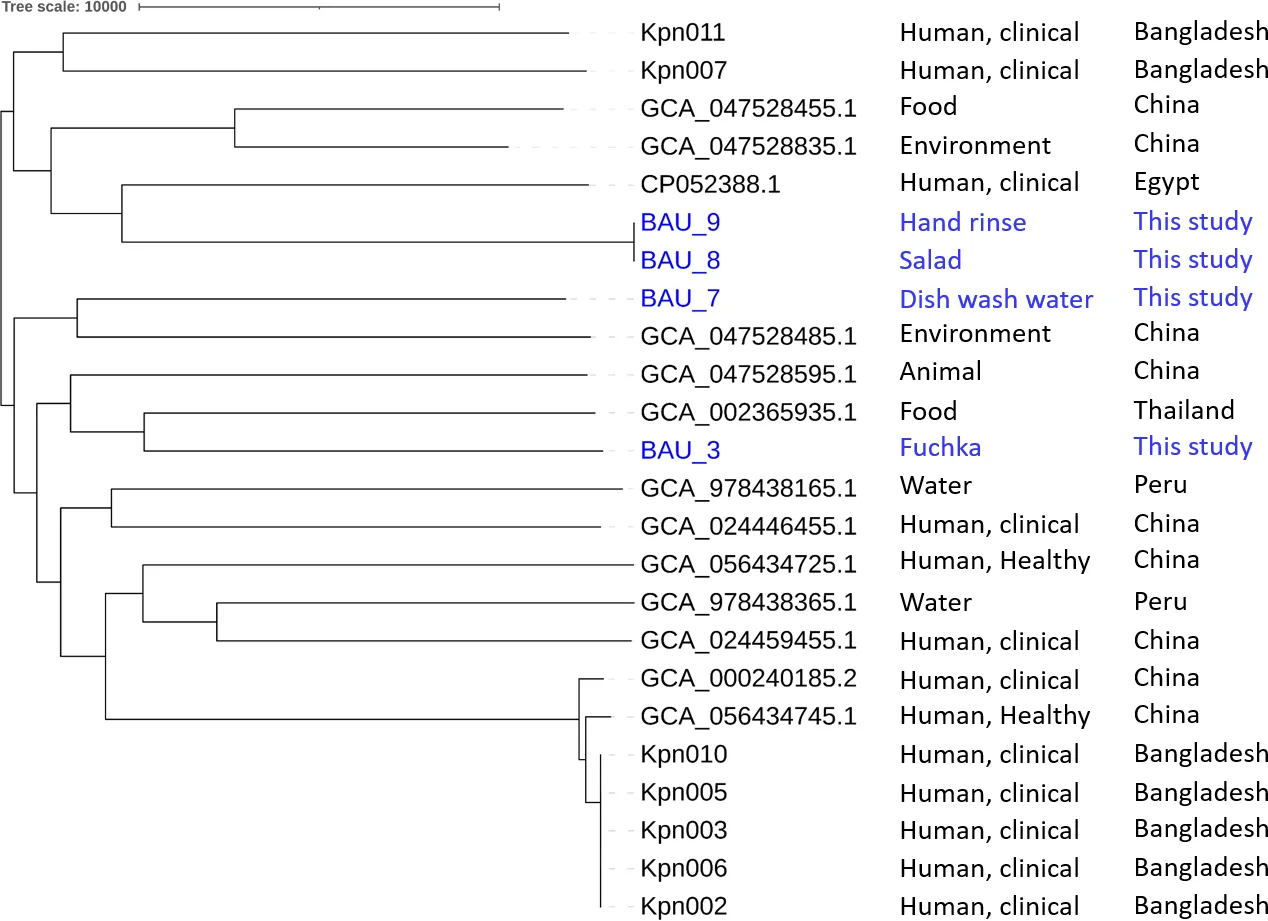
Phylogenomic relationships of *Klebsiella pneumoniae* isolates recovered from street food and food-handling environments. The maximum-likelihood tree was constructed from recombination-filtered core genome alignment using Gubbins and IQ-TREE. Isolates from the present study are highlighted in blue. Branch length represents nucleotide substitutions across the genome. Information on the right side indicates the host and source and the country of origin of the isolates.

Draft genome assemblies ranged from 5.24 to 5.36 Mb (GC content 57.31%–57.58%), consistent with published *K. pneumoniae* genomes. MPS_KP(S)_8 and MPS_KP(HW)_9 yielded highly contiguous assemblies (N50 = 355,520 bp; 36–38 contigs), while MPS_KP(DW)_7 (N50 = 148,197 bp) and MPS_KP(F)_3 (N50 = 37,664 bp) were comparatively less contiguous; no ambiguous bases were detected in any assembly (Table S1). All four isolates were confirmed as *Klebsiella pneumoniae* by Type Strain Genome Server (TYGS)-based phylogenomic species identification, clustering with reference *K. pneumoniae* strains (Fig. S1). Multi-locus sequence typing (MLST) assigned three sequence types: ST2938 (MPS_KP(F)_3), ST189 (MPS_KP(DW)_7), and ST2262 (MPS_KP(S)_8 and MPS_KP(HW)_9) (Table S2). Kaptive-based capsular typing identified K31-O5 (MPS_KP(F)_3), KL128-O3γ (MPS_KP(DW)_7), and K64-O2α (MPS_KP(S)_8 and MPS_KP(HW)_9). The KL128 locus represents a capsular type not previously reported in food-associated *K. pneumoniae* from South Asia. Plasmid replicon analysis identified the IncFIB(K) and pKP1433 replicons in ST2938 and an IncN replicon in ST189; no replicons were detected in either ST2262 isolate. IncFIB(K) is a hallmark of *K. pneumoniae* virulence plasmids, while IncN is a broad-host-range conjugative replicon associated with horizontal transfer of resistance determinants. Comprehensive Antibiotic Resistance Database (CARD) and ResFinder revealed multiple antibiotic resistance genes in the study isolates. Most of the resistance genes are associated with multidrug efflux, while isolate-specific *SHV β-lactamase* variants were identified, including *SHV*-11 in ST2938, *SHV*-80 in ST189, and *SHV*-108 in both of the ST2262 isolates. The presence of *SHV β-lactamase* correlates with the widespread resistance of the study isolates to β-lactam antibiotics (ampicillin, cephalosporins). In addition, the presence of *lpt*D, *oqx*A, and Kpn/*Klebsiella*-associated efflux determinants might contribute to the multidrug resistance of the *K. pneumoniae* isolates in this study (Table S3). Virulence profiling of the four WGS isolates revealed a conserved core virulome, with notable presence of a complete T6SS apparatus in two isolates (Fig. 13). Besides this, complete type I (*fim*A*–fim*K) and type III (*mrk*A*–mrk*J) fimbrial operons, iron acquisition systems (enterobactin pathway, *iro*E, *iro*N*, and iut*A), and type VI secretion system components were detected, consistent with the high prevalence of *wab*G, *uge*, and *mrk*D detected by PCR across the full isolate collection (Fig. 13). PathogenFinder predicted all four isolates as probable human pathogens. Core-genome phylogeny demonstrated that MPS_KP(S)_8 (BAU_8) and MPS_KP(HW)_9 (BAU_9) strains clustered with human clinical isolates from Egypt, while the MPS_KP(F)_3 strain (BAU_3) grouped with a food-derived isolate from Thailand, and the MPS_KP(DW)_7 strain (BAU_7) with environmental isolates from China (Fig. 14). The clustering of food- and food-handling environment-derived isolates with human clinical strains indicates that *K. pneumoniae* lineages circulating in fuchka and its associated environment share evolutionary ancestry with clinically important strains.

## DISCUSSION

Hospital-acquired infections caused by *K. pneumoniae* have increased markedly in recent years. Beyond its role in clinical settings, *K. pneumoniae* has emerged as a major contributor to the world AMR crisis due to its ability to acquire and disseminate resistance determinants. While extensive studies have focused on nosocomial infections and clinical isolates, the presence of *K. pneumoniae* in environmental settings and foodborne transmission routes remains insufficiently explored (2). Recent reports have identified hypervirulent and MDR *K. pneumoniae* in non-clinical niches including livestock, wastewater, surface soil, and companion animals, which significantly indicate its ecological versatility and zoonotic potential (27–29). Despite this, a significant knowledge gap remains regarding its presence in ready-to-eat foods, especially in street foods widely consumed in low- or middle-income countries. The detection of drug-resistant and virulent *K. pneumoniae* not only indicates poor hygiene and lack of sanitation during food handling niches, also highlights a potential dissemination route that has been neglected (25). Therefore, this study aimed to assess the prevalence and potential exposure pathway of *K. pneumoniae* in “fuchka,” the most popular and widely consumed RTE in Southeast and South Asian countries, including Bangladesh, as well as the determination of its drug resistance pattern and detection of virulence determinants.

A total of 42 *K. pneumoniae* strains were isolated from 90 fuchka samples and confirmed by PCR, MALDI-TOF, and 16S rRNA sequencing. Although this prevalence is lower than the 31.8% reported in street foods by Bedair et al. (30), it nevertheless demonstrates that fuchka acts as a reservoir for *K. pneumoniae* along with other enteric bacteria. Importantly, the overall distribution of isolates varied across sample sources, with street-vended fuchka yielding the highest number of isolates (*n* = 18), followed by fuchka shops (*n* = 12), while comparatively fewer isolates (*n* = 6) were obtained from restaurant and homemade delivery service samples. This variation in contamination levels reflects differences in hygienic practices, handling conditions, and environmental exposure across the sources (19). The higher recovery of *K. pneumoniae* from street vendors and small shops may be explained by limited access to clean water, inadequate hand- and utensil-washing facilities, and minimal enforcement of food safety regulations in these settings (31). Additionally, cross-contamination may occur through repeated handling, contact with contaminated surfaces, and use of raw ingredients such as onion, cucumber, and tomato, which are frequently added to fuchka without further processing. This interpretation is supported by Ashwath et al. (32), who reported the presence of *K. pneumoniae* in broiler meat and fresh vegetables, indicating that commonly used food ingredients can serve as entry points for this pathogen. In contrast, restaurant and homemade fuchka are typically prepared in relatively clean, confined, and hygienic environments, which may reduce contamination risk (33). Similar trends have been reported in other studies where *K. pneumoniae* was also reported in 55% of raw food and 15% of ready-to-eat food in Singapore, at varying frequencies depending on the level of hygiene control (24). Furthermore, Giri et al. (25) reported frequent detection of *K. pneumoniae* in panipuri, a food comparable to fuchka, particularly from street vendors and small shops with minimal hygiene practices. These findings support our observation and further emphasize the dissemination of *K. pneumoniae* within informal food settings.

PCR-based serotyping detected K3 (9.4%) and K5 (7.1%) in the isolates, while 83.3% were non-typeable. Though hypervirulent serotypes K1 and K2 were absent, the presence of K3 and K5 serotypes has been previously associated with extra-intestinal and invasive infections, including pneumonia and liver abscess, and has also been reported from food sources such as dairy products (27, 34). WGS-based serotyping of the four representative isolates revealed higher-resolution serotype assignments (K31, K128, K64), which do not contradict but complement PCR findings, as these analyses were performed on different isolates from the broader collection. K64, shared by the clonal pair MPS_KP(DW)_8 and MPS_KP(DW)_9, has been previously documented in clinical *K. pneumoniae* causing bloodstream infections and pneumonia, providing biological precedent for the public health concern raised by its detection in a food setting (35). The novel K128 locus in MPS_KP(DW)_7, with all 23 expected genes intact at near-perfect identity, represents a rare or previously undescribed capsular variant whose clinical significance warrants further investigation. Moreover, across both typeable and non-typeable isolates, virulence gene co-occurrence was consistently observed, indicating that pathogenic potential is not restricted to capsule-typeable strains, a similar pattern previously reported in clinical *K. pneumoniae* by Hasani et al. (34), who documented comparable virulence gene burdens across typeable and non-typeable isolates.

High prevalence of adhesion, biofilm, and capsule-associated genes such as *wab*G (90.5%), *uge* (50%), *mrk*D (52.4%), and *wca*G (47.8%) suggests a strong adaptive capacity and survival under adverse environmental conditions. These findings are consistent with niche-specific adaptation reported in food-derived isolates elsewhere (24). Universal carriage of *khe* and *uge* genes (100% to 95.2%) across all isolates in our study is consistent with previous reports, reinforcing their role as conserved virulence determinants in *K. pneumoniae* (32, 36). The absence of *rmpA* and *bfp* genes in our isolates is consistent with findings from other food-associated studies (24, 32). This may reflect a degree of attenuation in hypervirulent traits among food-borne *K. pneumoniae* compared to clinical strains (36), suggesting WGS virulence profiling confirmed and expanded upon these findings, revealing a conserved core virulome encompassing type I and III fimbriae (*fim*A-*fim*K*, mrk*A-*mrk*J), ECP pili (*ecp*A-*ecp*R), multiple iron acquisition systems, and capsule-regulatory genes. Most significantly, a complete T6SS apparatus with all 13 core components, including the essential effector delivery components hcp/tssD, vgrG/tssI, clpV/tssH, and the membrane-anchoring complex *dot*U/*tss*L and *icm*F/*tss*M, was detected in MPS_KP(DW)_7, MPS_KP(S)_8, and MPS_KP(HW)_9. The T6SS is a secretion system associated with interbacterial competition, host interaction, and gut colonization (37). Its complete form present in food-associated isolates, including the clinically relevant ST2262 isolate MPS_KP(DW)_8, suggests that these strains may contribute to persistence and host interaction following ingestion.

The antibiotic susceptibility pattern of *K. pneumoniae* strains revealed a critical insight into potential treatment challenges posed by these foodborne strains. Universal ampicillin resistance is intrinsic to *K. pneumoniae*, indicating complete inefficacy of this commonly used β-lactam antibiotic (38). High cefixime (52.4%) resistance suggests ESBL-producing strains are circulating in food environments. These findings are further supported at the genomic level by detection of *bla*_SHV_ variants (*bla*_SHV_-11, *bla*_SHV_-80, *bla*_SHV_-108) in WGS-sequenced isolates. The agreement between the phenotypic resistance data and the WGS findings strengthens confidence in both approaches, while WGS provided the added advantage of identifying exactly which *bla*_SHV_ variant was present in each isolate, something phenotypic testing alone cannot determine. Comparable findings were observed by Bedair et al. (30), where *K. pneumoniae* strains from street food sources demonstrated 100% resistance to cefuroxime and cefradine and 98.7% resistance to amoxicillin-clavulanic acid. Besides β-lactam resistance, PCR screening detected several other resistance genes across the isolates, including *tet*A (56.7%), *aac*(6′) (35.7%), *sul*1 (33.3%), and *int*2 (7.1%), indicating resistance potential across tetracycline, aminoglycoside, and sulfonamide classes. WGS further revealed the presence of efflux pump genes *oqx*A, *lpt*D, and other *Klebsiella*-associated determinants, which likely contribute to resistance beyond what the targeted PCR panel was designed to detect (39). The presence of *int*2, though found in a smaller proportion of isolates, is worth noting because class 2 integrons are known to facilitate the capture and spread of new resistance genes, meaning these strains may have the capacity to become more resistant over time (40). Overall, 35.7% of these isolates were MDR, which is broadly comparable to Bedair et al. (30), who reported 27.3% of *K. pneumoniae* from ready-to-eat street food in Egypt and is consistent with reports of increasing MDR *Klebsiella* in food environments across low- and middle-income countries (32, 41). Despite these resistance concerns, high susceptibility was retained for nitrofurantoin and trimethoprim-sulfamethoxazole (100%), fosfomycin (97.6%), imipenem (90.5%), and ciprofloxacin (92.7%), suggesting these agents may retain therapeutic utility. However, the detection of *oqx*A, a plasmid-carried gene associated with reduced fluoroquinolone susceptibility in the genomic data, is a reminder that current susceptibility does not guarantee future utility, and these strains should be monitored closely (39, 42). Together, these findings suggest that street food environments may play a quiet but important role in harboring and potentially spreading clinically relevant resistant *K. pneumoniae* in the community (30).

Biofilm production of *K. pneumoniae* isolates was observed in 31% of isolates and was significantly associated with the MDR phenotype and the MAR index (*P* < 0.01). This observation is consistent with the recognized role of the biofilm matrix in reducing antibiotic penetration and promoting resistance gene transfer (43). These findings align with reports in other food-associated *K. pneumoniae* studies (41, 44).

PCR-based resistance gene profiling across all 42 isolates revealed *int*1 as the most prevalent determinant (83.3%), indicating substantial potential for horizontal resistance gene transfer (45). The high co-occurrence of *int*1 with virulence genes (>90%) suggests possible co-mobilization of virulence and resistance determinants on integron-associated mobile elements, consistent with Yakout and Ali (46). Co-occurrence analysis identified several statistically significant associations: *wcaG–sul1* (50%, *P* = 0.02), *fimH–aac*6′, and *ybtS–sul1* (100%, *P* = 0.01), suggesting functional linkage between adhesion determinants and aminoglycoside and sulfonamide resistance. Similar co-occurrence patterns between the virulence traits and resistance determinants of *K. pneumoniae* isolates have been reported by El-Domany et al. (47) in Egypt and Hu et al. (48) in China. The WGS data provide direct genomic evidence that *K. pneumoniae* recovered from fuchka and its food-handling environment harbor clinically relevant characteristics. ST189, identified in MPS_KP(DW)_7 (BAU_7) (dishwashing water), is a globally distributed clinical sequence type previously associated with bacteremia and urinary tract infections (49). The K64-O2α serotype shared by MPS_KP(S)_8 and MPS_KP(HW)_9 (BAU_8 and BAU_9) has been documented in clinical *K. pneumoniae* causing serious infections, and these isolates clustered phylogenomically with human clinical strains from Egypt, providing genomic evidence suggesting that food-associated lineages in this study share evolutionary ancestry with strains implicated in human disease. This finding is independently corroborated by PathogenFinder, which predicted all four isolates as probable human pathogens. Plasmid-borne IncFIB(K) and pKP1433 replicons in the fuchka-derived MPS_KP(F)_3, and an IncN replicon in MPS_KP(DW)_7, indicate that these strains carry mobile genetic platforms capable of horizontally disseminating resistance and virulence determinants within the food environment and beyond (50). The novel KL128 capsular locus identified in MPS_KP(DW)_7 expands the known diversity of food-associated *K. pneumoniae* capsular types in South Asia. Collectively, these genomic findings elevate the public health concern from phenotypic observation to molecular evidence; fuchka and its associated food-handling environments harbor *K. pneumoniae* strains genomically related to human clinical isolates, carrying mobile resistance elements and conserved core virulence architecture, warranting their integration into One Health surveillance frameworks for antimicrobial-resistant pathogens.

These findings underscore the need to integrate street foods into national antimicrobial resistance surveillance programs and strengthen hygiene training and regulatory oversight of ready-to-eat food sellers. However, the study is limited by its restricted geographic scope, modest sample size, and lack of functional virulence assay transmission dynamics. Future research should therefore adopt multi-regional and longitudinal sampling, expanded WGS for all isolates, functional virulence assays, and quantitative risk assessment approaches to better elucidate the role of street foods in the dissemination of antimicrobial-resistant *K. pneumoniae*.

## MATERIALS AND METHODS

### Sampling

A total of 90 fuchka samples, including fuchka shells, toppings, tamarind sauce, salad, dishwash water, and hand rinse water of sellers, were collected from different sources in Mymensingh, Bangladesh. Samples were obtained from four types of outlets: street vendors, fuchka-selling shops, restaurants, and homemade delivery food services. Samples were collected aseptically, transported in an ice box, and processed within the same day.

### Isolation of *Klebsiella pneumoniae*

Fuchka, including the fillings and toppings, was homogenized, and 10 g was suspended in 90 mL of nutrient broth. The suspension was then incubated at 37°C for 24 h for primary enrichment. Then, a 10 μL loopful of the enriched suspension was streaked onto MacConkey agar (Himedia, India) and incubated at 37°C for 24 h. Colonies exhibiting large, mucoid, pink morphology indicative of *K. pneumoniae* were selected. Subsequent sub-culture was performed to obtain pure culture for further characterization.

### Identification of *Klebsiella pneumoniae*

Presumptive isolates were initially characterized using Gram staining, followed by a series of standard biochemical assays, including urease activity, indole test, carbohydrate fermentation test, motility assessment, and citrate utilization by following Patra et al. (51). Molecular confirmation was performed by PCR targeting the *rpo*B gene using previously described primers (52). In addition, species identification was further validated using MALDI-TOF MS (matrix-assisted laser desorption ionization time-of-flight mass spectrometry) and 16S rRNA sequencing. A score >2.0 was considered reliable for species-level identification (Table S4).

### Genomic DNA extraction

For genomic DNA extraction of all the isolates, a simple boiling and thawing method (53) was explored, with slight modifications. Briefly, 1 mL of cultured broth was transferred into an Eppendorf tube and centrifuged at 5,000 rpm for 5 min. The supernatant was then discarded, and the pellet was re-suspended with 500 μL sterile distilled water. After thorough vortexing, the suspension was boiled at 100°C for 10 min and then immediately chilled on ice for 10–15 min to stabilize DNA. The lysate was centrifuged at 10,000 rpm for 10 min to remove the cellular debris, and 250 μL of supernatant was collected in a sterile 1.5 mL Eppendorf tube for use as a DNA template. The quantity and purity of DNA were assessed by Nanodrop One (Thermo Fisher Scientific, USA).

### Detection of virulence and resistance genes

All confirmed isolates were screened by PCR to detect the virulence genes, such as *fim*H, *ybt*A, *mrk*D, *wab*G, *wca*G, *rmp*A, *khe*, *ure*A, *uge*, *bfp*A, and *aerobactin,* as well as to determine the capsular serotypes, *viz*., K1, K2, K3, K5_wzx, and K20. Isolates were also screened for the detection of specific antibiotic resistance determinants (*aac*6′, *aad*A2, *tet*A, *tet*B, *tet*C, *sul*1, *int*1, *int*2) using previously reported primers. Primer sequences, expected amplicon sizes, and thermal conditions are provided in Table 4.

**TABLE 4:** Target genes, oligonucleotide sequences, and thermal conditions used for PCR

	Target gene	Primer sequence (5′−3′)	Thermal condition					No. of cycles	Amplicon size (bp)	Ref
			Initial denaturation	Denaturation	Annealing	Elongation	Final elongation			
	ERIC	ATGTAAGCTCCTGGGGATTCAC AAGTAAGTGACTGGGGTGAGCG	94°C, 5 m	94°C, 1 m	49°C, 1 m	72°C, 3 m	72°C, 10 m	35		(54)
Virulence genes	*wab*G	CGGACTGGCAGATCCATATC ACCATCGGCCATTTGATAGA	94°C, 3 m	94°C, 45 s	54°C, 45 s	72°C, 45 s	72°C, 7 m	35	683	(55)
	*wca*G	GGTTGGKTCAGCAATCGTA ACTATTCCGCCAACTTTTGC	95°C, 5 m	94°C, 30 s	55°C, 30 s	72°C, 1.5 m	72°C, 10 m	35	169	(56)
	*fim*H	TGCTGCTGGGCTGGTCGATG GGGAGGGTGACGGTGACATC	94°C, 3 m	94°C, 45 s	55°C, 45 s	72°C, 45 s	72°C, 7 m	35	550	(55)
	*ure*	GCTGACTTAAGAGAACGTTATG GATCATGGCGCTACCTTA	94°C, 3 m	94°C, 45 s	54°C, 45 s	72°C, 45 s	72°C, 7 m	35	428	(57)
	*rmp*A	ACTGGGCTACCTCTGCTTCA CTTGCATGAGCCATCTTTCA	94°C, 5 m	94°C, 1 m	54°C, 1 m	72°C, 1 m	72°C, 7 m	35	516	(58)
	*mrk*D	AAGCTATCGCTGTACTTCCGGCA GGCGTTGGCGCTCAGATAGG	94°C, 5 m	94°C, 30 s	58°C, 30 s	72°C, 1 m	72°C, 7 m	35	340	(32)
	*ybt*A	ATGACGGAGTCACCGCAAAC TTACATCACGCGTTTAAAGG	94°C, 3 m	94°C, 30 s	50°C, 30 s	72°C, 30 s	72°C, 5 m	35	960	(59)
	*khe*	TGATTGCATTCGCCACTGG GGTCAACCCAACGATCCTG	94°C, 5 m	94°C, 45 s	55°C, 40 s	72°C, 1 m	72°C, 5 m	35	428	(32)
	*uge*	GATCATCCGGTCTCCCTGTA TCTTCACGCCTTCCTTCACT	94°C, 5 m	94°C, 30 s	53°C, 30 s	72°C, 1 m	72°C, 10 m	35	534	(55)
	*aerobactin*	GCATAGGCGGATACGAACAT CACAGGGCAATTGCTTACCT	94°C, 5 m	94°C, 30 s	55°C, 30 s	72°C, 1 m	72°C, 5 m	35	556	(58)
	*bfp*	AATGGTGCTTGCGCTTGCTGC GCCGCTTTATCCAACCTGGTA	94°C, 5 m	94°C, 30 s	58°C, 30 s	72°C, 1 m	72°C, 5 m	35	336	(32)
Serotype	K1	AGATAGAGGTGTATTGTCGC GAGCTCTATATGTTGGATGC	94°C, 5 m	94°C, 45 s	52°C, 45 s	72°C, 45 s	72°C, 5 m	35	321	(60)
	K2	TCATACTTGACAGAGGGAGTAG ACGATCGTTACAGTGACAAG	94°C, 5 m	94°C, 45 s	54°C, 45 s	72°C, 45 s	72°C, 5 m	35	352	
	K3	TAGGCAATTGACTTTAGGTG AGTGAATCAGCCTTCACCT	94°C, 5 m	94°C, 30 s	58°C, 90 s	72°C, 90 s	72°C, 10 m	35	280	(58)
	K5	TGGTAGTGATGCTCGCGA CCTGAACCCACCCCAATC	94°C, 5 m	94°C, 30 s	58°C, 90 s	72°C, 90 s	72°C, 10 m	35	549	
	K20	CGGTGCTACAGTGCATCATT GTTATACGATGCTCAGTCGC	94°C, 5 m	94°C, 30 s	58°C, 90 s	72°C, 90 s	72°C, 10 m	35	741	(56)
Resistance genes	*aac*6*′*	TTGCGATGCTCTATGAGTGGCTA CTCGAATGCCTGGCGTGTTT	94°C, 4 m	94°C, 45 s	55°C, 40 s	72°C, 45 s	72°C, 7 m	34	482	(61)
	*aad*A2	TGTTGGTTACTGTGGCCGTA GATCTCGCCTTTCACAAAGC	94°C, 4 m	94°C, 45 s	54°C, 40 s	72°C, 45 s	72°C, 7 m	34	623	(62)
	*sul*1	TTTCCTGACCCTGCGCTCTAT GTGCGGACGTAGTCAGCGCCA	94°C, 4 m	94°C, 45 s	55°C, 40 s	72°C, 45 s	72°C, 7 m	32	425	(63)
	*tet*A	GCTACATCCTGCTTGCCTTC CATAGATCGCCGTGAAGAGG	95°C, 5 m	95°C, 30 s	60°C, 1 m	72°C, 1 m	72°C, 7 m	35	210	(64)
	*tet*B	TTGGTTAGGGGCAAGTTTTG GTAATGGGCCAATAACACCG	95°C, 5 m	95°C, 30 s	60°C, 1 m	72°C, 1 m	72°C, 7 m	35	359	
	*tet*C	CTTGAGAGCCTTCAACCCAG ATGGTCCTCATCTACCTGCC	95°C, 5 m	95°C, 30 s	60°C, 1 m	72°C, 1 m	72°C, 7 m	35	418	
	*int*1	GGGTCAAGGATCTGGATTTCG ACATGCGTGTAAATCATCGTCG	94°C, 5 m	94°C, 30 s	62°C, 30 s	72°C, 1 m	72°C, 7 m	30	483	(65)
	*int*2	CACGGATATGCGACAAAAAGGT GTAGCAAACGAGTGACGAAATG	94°C, 5 m	94°C, 30 s	62°C, 30 s	72°C, 1 m	72°C, 7 m	30	788	

### Quantitative biofilm formation assay

The biofilm formation ability of the isolates was assessed using a 96-well microtiter plate assay following a previously described method with minor modifications (66). Isolates were cultured in LB broth at 37°C for 24 h. A sterile 96-well plate was prepared by adding 198 μL of Trypticase Soy Broth (TSB) supplemented with 1% glucose and 2 μL of overnight LB culture. Negative controls contained only sterile TSB. Plates were incubated for 24 h at 37°C, after which the contents were discarded by inversion and gently tapped onto absorbent paper. Wells were washed four times with 200 μL of phosphate-buffered saline (pH 7.2) to remove planktonic cells. Two hundred microliters of 0.1% crystal violet in water was used to stain the wells for 15 min at room temperature, and then rinsed four times with distilled water to remove excess stain. Subsequently, 200 μL of 95% ethanol was added to each well to solubilize the biofilm for 10 to 15 min at room temperature. Absorbance was measured at 570 nm using an ELISA plate reader (Thermo Fisher Scientific, USA), with wells containing only ethanol serving as blanks. Each assay was performed twice, and mean values were calculated for each isolate. The cut-off optical density for biofilm formation was defined as three standard deviations above the mean OD of the negative control. Based on OD values, isolates were categorized into non-, weak, moderate, or strong biofilm producers according to established criteria (67).

### ERIC-PCR

ERIC-PCR was performed using the primers listed in Table 4, with thermal cycling carried out on a Bio-Rad PTC-200 Thermal Cycler (Bio-Rad). The specific banding pattern was then observed and analyzed by GelJ version 2.0 (68). The resulting profiles were evaluated and clustered using the Dice similarity coefficient and the neighbor-joining method to build the phylogenetic tree. Isolates having two or more different specific bands were classified as different ERIC types. The dendrogram was made according to the cluster grouping (68).

### PCR amplification

All PCRs were performed in a total volume of 25 µL, each containing 1.0 U of Taq DNA polymerase (Takara Bio, Japan), 1.0 µM of each primer, 2.5 mM MgCl2, 0.2 mM of each dNTP, and 40 ng of genomic DNA template. Thermocycling conditions of PCR amplification for all used primers are mentioned in Table 4. Amplified PCR products were then separated on 1.5% agarose gel (Lonza SeaKem LE Agarose, Lonza, Basel, Switzerland) in 1× TAE buffer (40 mM Tris-HCl, 1.18 mL acetic acid, 2 mM EDTA, pH 8.0) containing Safe Red DNA stain (Hebei SanshiBio-Tech Co. Ltd., China), at a constant voltage of 100 V for 30 min for electrophoresis, and the banding patterns were visualized and photographed using the Bio-Rad GelDoc Go Imaging System (Bio-Rad Laboratories Private Limited, Germany).

### Antibiotic susceptibility testing and MDR analysis

Antibiotic susceptibility tests for all confirmed isolates were carried out by adopting the Kirby-Bauer disk diffusion method by following the Clinical and Laboratory Standards guidelines (69). Nine antibiotics (Oxoid, Germany) were evaluated: ampicillin (10 μg), cefixime (5 μg), nitrofurantoin (300 μg), fosfomycin (30 μg), ciprofloxacin (5 μg), gentamicin (10 μg), imipenem (10 μg), tetracycline (30 μg), and sulfamethoxazole-trimethoprim (25 μg). All the *K. pneumoniae* isolates were grouped as resistant and susceptible based on their diameters of inhibition zones. *Escherichia coli* strain ATCC 25,922 was used as a reference strain. The MDR patterns were determined following the criteria (resistant against at least one agent of ≥3 antibiotic classes) of Magiorakos et al. (70), and the MAR index was calculated as per Krumperman (71), defined as the ratio of the number of antibiotics to which an isolate exhibited resistance to the total number of antibiotics tested.

### Sequencing and phylogenetic analysis

16S rDNA genes, from three representative isolates of *Klebsiella pneumoniae,* were amplified by PCR using the primer sets (F: 5′-AGAGTTTGATCCTGGCTCAG-3′; R: 5′-CTTGTGCGGGCCCCCGTCAATTC-3′), confirmed by gel electrophoresis, and purified using the QIAamp PCR purification kit (QIAGEN GmbH, Hilden, Germany). Sanger dideoxy sequencing was performed by BD Genomes Laboratory, Savar, Dhaka. Both forward and reverse reads were assembled into a consensus sequence using CLC Sequence Viewer v.8 (http://www.clcbio.com) and confirmed by BLAST. For the phylogenetic analysis tree, 33 reference 16S rRNA sequences of *K. pneumoniae* were downloaded from GenBank (https://www.ncbi.nlm.nih.gov/nucleotide/), aligned using MUSCLE in MEGA11, and a maximum-likelihood tree was constructed (TN93+G model, 1,000 bootstrap replicates) and annotated using iTOL v.5.

### Whole-genome sequencing and genomic characterization

Four representative *K. pneumoniae* isolates: BAU_3 (strain MPS_KP(F)_3) from fuchka, BAU_7 (strain MPS_KP(DW)_7) from dishwashing water, BAU_8 (strain MPS_KP(S)_8) from salad, and BAU_9 (strain MPS_KP(HW)_9) from vendor hand-rinse water were subjected to whole-genome sequencing. Genomic DNA was extracted using the QIAamp DNA Blood Mini Kit (QIAGEN), quantified by Qubit (Q32851, Thermo Fisher Scientific), and sequenced on the Illumina NextSeq2000 platform (150 bp paired-end) using the NEBNext Ultra II DNA Library Prep Kit for Illumina (E7103, New England BioLabs). Raw reads were quality-filtered with fastp (v.0.23.2) and assembled *de novo* using Unicycler (v.0.5.1); assembly quality was assessed with QUAST (v.5.3.0), and genomes were annotated with Prokka (v.1.11.x). Species identity was confirmed using the TYGS (https://tygs.dsmz.de). MLST was performed using the BIGSdb Pasteur (https://bigsdb.pasteur.fr/klebsiella/) *Klebsiella* scheme (seven housekeeping loci). Capsular (K) and O-antigen loci were assigned using the Kaptive web platform (https://kaptive-web.erc.monash.edu) (72, 73). Plasmid replicons were identified with PlasmidFinder available at the Center for Genomic Epidemiology (https://cge.food.dtu.dk/services/PlasmidFinder/). Virulence genes were detected by DIAMOND BLASTP against VFDB set B (≥90% identity and coverage), and antimicrobial resistance genes were identified using the CARD online (https://card.mcmaster.ca/). Pathogenic potential was assessed with PathogenFinder (https://cge.food.dtu.dk/services/PathogenFinder/). Pangenome analysis was conducted with Panaroo (v.1.3.x; core genome ≥99% presence) incorporating 20 publicly available *K. pneumoniae* reference genomes. A maximum-likelihood phylogeny was reconstructed from recombination-filtered core-genome SNPs using Gubbins (v.3.4.x) and IQ-TREE and visualized on iTOL.

### Statistical analysis

Statistical analysis was done using SPSS IBM software version 26.0 to determine significant relationships between the virulence determinants and antibiotic resistance genes. A χ^2^ test was used to estimate the *P*-value of the co-occurrence percentage of the virulence and resistance genes and the association between the biofilm category and MDR. A *P*-value of <0.05 was considered statistically significant.

### Conclusion

To our knowledge, this study is the first report in Bangladesh to demonstrate that fuchka, the widely consumed street food, can serve as a reservoir of virulent and MDR *K. pneumoniae*. Whole-genome sequencing further revealed that food-associated lineages in this study share phylogenomic ancestry with human clinical strains, elevating these findings from phenotypic observation to molecular evidence of public health concern. These findings suggest that *K. pneumoniae* may not be confined only to clinical settings but also circulate through the informal food chain, posing a potential risk of community-acquired infections caused by antimicrobial-resistant strains. From a practical standpoint, targeted hygiene training for street food vendors, improved access to clean water, and the inclusion of informal food sectors in national antimicrobial surveillance programs are urgently needed to reduce exposure risks. Future research should move beyond culture-based approaches to incorporate metagenomic analyses, quantitative exposure assessments, and longitudinal surveillance to better characterize transmission dynamics, microbial community interactions, and the true public health burden associated with street-vended foods.

## Data Availability

The whole-genome sequencing data of the four *K. pneumoniae* isolates characterized in this study have been deposited in NCBI GenBank under BioProject accession number PRJNA1463975. The genome assemblies are available under BioSample accession numbers SAMN59651338 (MPS_KP(F)_3), SAMN59651989 (MPS_KP(DW)_7), SAMN59651995 (MPS_KP(S)_8), and SAMN59652000 (MPS_KP(HW)_9) and genome assembly accession numbers JBYFKM000000000, JBYFKN000000000, JBYFKO000000000, and JBYFKP000000000.

## SUPPLEMENTAL MATERIAL

Fig. S1 aem.00996-26-s0001.tif Whole genome-based identification of the *K. pneumoniae* strains isolated in this study. The genome sequence in FASTA format was uploaded as an input, and a comparative type strain-based tree was constructed through Type (Strain) Genome server (TYGS) (https://tygs.dsmz.de/). Type strain-based analysis identified all the isolates (red square nodes) from this study as *K. pneumoniae*.

Supplemental tables aem.00996-26-s0002.docx Tables S1 to S4.

ASM does not own the copyrights to Supplemental Material that may be linked to, or accessed through, an article. The authors have granted ASM a non-exclusive, world-wide license to publish the Supplemental Material files. Please contact the corresponding author directly for reuse.
