# Supplementary material for: Klebsiella pneumoniae from fuchka: insights into the genetic diversity, virulence, and antibiogram profile
Source: Appl Environ Microbiol. 2026 Aug 5;92(9):e00996-26. doi: 10.1128/aem.00996-26 (PMC13599500; doi:10.1128/aem.00996-26)
Supplement: Supplementary file 1 [file aem.00996-26-s0002.docx]

**Table S1:** Characteristics of the assembled *K. pneumoniae* genomes

| **Assembly** | **MPS_KP(F)_3** | **MPS_KP(DW)_7** | **MPS_KP(S)_8** | **MPS_KP(HW)_9** |
| --- | --- | --- | --- | --- |
| BioSample accession | SAMN59651338 | SAMN59651989 | SAMN59651995 | SAMN59652000 |
| Genome assembly accession | JBYFKM000000000 | JBYFKN000000000 | JBYFKO000000000 | JBYFKP000000000 |
| # contigs | 285 | 88 | 36 | 38 |
| Largest contig | 175921 | 336694 | 768485 | 556949 |
| Total length | 5252570 | 5355242 | 5245643 | 5246289 |
| GC (%) | 57.31 | 57.36 | 57.58 | 57.58 |
| N50 | 37664 | 148197 | 355520 | 355520 |
| N90 | 7676 | 41202 | 141781 | 100212 |
| L50 | 32 | 14 | 6 | 6 |
| L90 | 154 | 37 | 14 | 15 |
| # N's per 100 kbp | 0 | 0 | 0 | 0 |

**Table S2**: MLST allelic profiles of *K. pneumoniae* isolates.

| **Strain name** | **ST** | ***gap*A** | ***inf*B** | ***mdh*** | ***pgi*** | ***pho*E** | ***rpo*B** | ***ton*B** |
| --- | --- | --- | --- | --- | --- | --- | --- | --- |
| MPS_KP(F)_3 | ST2938 | 2 | 4 | 2 | 1 | 7 | 4 | 12 |
| MPS_KP(DW)_7 | ST189 | 2 | 3 | 41 | 1 | 17 | 4 | 46 |
| MPS_KP(S)_8 | ST2262 | 2 | 4 | 1 | 26 | 7 | 27 | 9 |
| MPS_KP(HW)_9 | ST2262 | 2 | 4 | 1 | 26 | 7 | 27 | 9 |

**Table S3:** Genomic characteristics of four *K. pneumoniae* isolates from fuchka.

| **Strain Name** | **ST** | **K-type** | **O-type** | ***bla*_SHV_ variant** | **Other AMR genes** | **T6SS** | **Key virulence** |
| --- | --- | --- | --- | --- | --- | --- | --- |
| MPS_KP(F)_3 | ST2938 | K31 | O5 | *bla*_SHV_-11 | *oqx*A, *fos*A_5, *kpn*F*, kpn*E  *lpt*D | Absent | *fim, mrk, ent, fep, iro, iut*A |
| MPS_KP(DW)_7 | ST189 | KL128 (novel) | O3γ | *bla*_SHV_-80 | *oqx*A, *oqx*B, *fos*A_3, *lpt*D, | Present | *fim, mrk, ecp, ent, fep, iro, iut*A |
| MPS_KP(S)_8 | ST2262 | KL64 | O2α | *bla*_SHV_-108 | *oqx*A, o*qx*B, *fos*A_6, *kpn*F, *lptD, mdtQ* | Present | *fim, mrk, ent, fep, iro, acr*AB, *rcs* |
| MPS_KP(HW)_9 | ST2262 | KL64 | O2α | *bla*_SHV_-108 | *oqx*A, *oqx*B, *fos*A_6, *kpn*F, *lptD, mdtQ* | Present | *fim, mrk, ent, fep, iro, acr*AB, *rcs* |

**Table S4:** MALDI-tof-MS value of tested *K. pneumoniae* isolates

| **Isolate ID** | **MALDI-tof-MS value** |
| --- | --- |
| 2(3) | 2.28 |
| 3(19) | 2.33 |
| 7(2) | 2.21 |
| 8(4) | 2.35 |
| 8(5) | 2.33 |
| 8(6) | 2.33 |
| 8(15iii) | 2.35 |
| 9(7) | 2.33 |
| 10(12) | 2.23 |
| 10(8) | 2.25 |
| 12(4) | 2.21 |
| 12(5) | 2.35 |
| 13(4) | 2.33 |
| 13(7) | 2.28 |
| 15(1) | 2.21 |
